# Diversity and Geographical Structure of *Xanthomonas citri* pv. *citri* on Citrus in the South West Indian Ocean Region

**DOI:** 10.3390/microorganisms9050945

**Published:** 2021-04-27

**Authors:** Olivier Pruvost, Damien Richard, Karine Boyer, Stéphanie Javegny, Claudine Boyer, Frédéric Chiroleu, Pierre Grygiel, Evelyne Parvedy, Isabelle Robène, Véronique Maillot-Lebon, Azali Hamza, Kanta Kumar Lobin, Marc Naiken, Christian Vernière

**Affiliations:** 1 CIRAD, UMR PVBMT, F-97410 Saint Pierre, La Réunion, France; richarddamienfr@gmail.com (D.R.); karine.boyer@cirad.fr (K.B.); stephanie.javegny@cirad.fr (S.J.); claudine.boyer@cirad.fr (C.B.); frederic.chiroleu@cirad.fr (F.C.); pierre.grygiel@orange.fr (P.G.); evelyneparvedy@gmail.com (E.P.); isabelle.robene@cirad.fr (I.R.); marie-veronique.maillot@cirad.fr (V.M.-L.); christian.verniere@cirad.fr (C.V.); 2 ANSES, Plant Health Laboratory, F-97410 St Pierre, La Réunion, France; 3 UFR Sciences et Technologies, Université de la Réunion, UMR PVBMT, F-97490 St Denis, La Réunion, France; 4 INRAPE, Moroni BP 289, Comoros; abdouazalihamza@gmail.com; 5 FAREI, Le Réduit 80835, Mauritius; lobinrajiv@gmail.com; 6 National Biosecurity Agency, Victoria P.O Box 464, Mahé, Seychelles; ceo@nba.gov.sc; 7 PHIM Plant Health Institute, CIRAD, INRAE, Institut Agro, IRD, University Montpellier, F-34398 Montpellier, France

**Keywords:** molecular epidemiology, citrus, Asiatic canker, copper resistance, aggressiveness

## Abstract

A thorough knowledge of genotypic and phenotypic variations (e.g., virulence, resistance to antimicrobial compounds) in bacteria causing plant disease outbreaks is key for optimizing disease surveillance and management. Using a comprehensive strain collection, tandem repeat-based genotyping techniques and pathogenicity assays, we characterized the diversity of *X. citri* pv. *citri* from the South West Indian Ocean (SWIO) region. Most strains belonged to the prevalent lineage 1 pathotype A that has a wide host range among rutaceous species. We report the first occurrence of genetically unrelated, nonepidemic lineage 4 pathotype A* (strains with a host range restricted to Mexican lime and related species) in Mauritius, Moheli and Réunion. Microsatellite data revealed that strains from the Seychelles were diverse, grouped in three different clusters not detected in the Comoros and the Mascarenes. Pathogenicity data suggested a higher aggressiveness of strains of one of these clusters on citron (*Citrus medica*). With the noticeable exception of the Comoros, there was no sign of recent interisland movement of the pathogen. Consistent with this finding, the *copL* gene, a marker for the plasmid-borne *copLAB* copper resistance that was recently identified in Réunion, was not detected in 568 strains from any islands in the SWIO region apart from Réunion.

## 1. Introduction

Emerging and invasive plant pests and pathogens are a major threat to food security and the sustainability of agricultural cash crop industry [1,2]. Emerging diseases are diseases that occur in new areas (i.e., geographical expansion of preadapted pests and pathogens), on new hosts (i.e., host jumps or changes in host range) or that present new traits (e.g., acquired resistance to antimicrobials) [3]. Geographical expansion is a major factor in the emergence of plant pathogens [4]. The rapid increase in the geographical expansion of preadapted pests and pathogens over the last century is undoubtedly linked to the globalization of international trade and the increase of international travel of humans [5,6].

Collectively, citrus is the most important fruit crop in terms of world production. Several major bacterial, fungal and viral pathogens, which usually figure on quarantine or action lists, represent a major challenge to citrus industries. Preventing their establishment or delaying their spread is a priority. *Xanthomonas citri* pv. *citri* (synonym = *X. citri* subsp. *citri*) causes Asiatic citrus canker (ACC). This bacterium originated from Asia (i.e., the center of origin of *Citrus*) [7] and has spread globally due to the transport of citrus plant material over long distances [8]. *X. citri* pv. *citri* induces canker-like lesions on the external tissues of leaf, fruit and stem. Severe infections cause premature fruit drop, defoliation and twig dieback, resulting in yield loss and export market restrictions. Infection occurs through natural openings (i.e., stomata) or wounds. Stomatal infections only occur on actively growing aerial organs of citrus, whereas wounds are susceptible to infection over much larger time frames [9,10].

Strains of *X. citri* pv. *citri* differ in host range and defense reaction types on nonhost species, which determines the pathotype classification. The host range of pathotypes A* and A^w^ is limited to certain acid citrus types. They have primarily been sampled from natural infections on Mexican lime (*Citrus x aurantiifolia*) [11,12]. In contrast, pathotype A strains infect nearly all *Citrus* species, as well as other rutaceous genera [8]. Molecular diagnostics and genotyping techniques are essential for efficient disease management in agroecosystems [13]. Minisatellite and microsatellite genotyping, respectively, are well suited for global and small-scale studies on the molecular epidemiology of *X. citri* pv. *citri* [14,15,16,17]. In general, they are congruent with SNP-based phylogenies [15,18]. Pathotype A strains are split into two lineages (lineage 1 and 2), while pathotype A^w^ and A* were each assigned to a single lineage (lineage 3 and 4, respectively) [15]. Pathotypes A^w^ and A* globally have a more restricted geographical distribution and have less impact on agriculture because of their narrow host range, although severe outbreaks have been reported in countries where their host species are grown widely [19,20,21]. Lineage 1 strains were largely responsible for the major geographical expansion of *X. citri* pv. *citri* in the 20th century and for most worldwide outbreaks [15]. Recently reported, the agricultural significance of lineage 2 strains has not been widely documented, although they are present in the pathogen’s area of origin and are expanding in West Africa [15,16].

ACC control is primarily based on eradication or integrated management. The latter involves a combination of measures. These include the use of disease-free, partially-resistant nursery plants; efficient windbreaks; the use of drip irrigation instead of overhead irrigation; the extensive application of copper compounds (or use of antibiotics, which is less frequent) when plant organs are most susceptible to infection and pruning of diseased shoots during grove maintenance operations [8,22]. The impact of ACC is host-, environment- and bacterial lineage-dependent. Bacterial spread and infection are primarily driven by the use of *X. citri* pv. *citri*-contaminated nursery plants for grove establishment but also by extreme weather events [14,16]. Another factor promoting infection is resistance to antibacterial copper compounds, which reduces the effectiveness of ACC control [23,24]. To date, copper resistance is mostly restricted to lineage 1 strains found in three areas: South America (Argentina), the Caribbean region (Martinique) and the South West Indian Ocean (SWIO) region (Réunion). Copper-resistant *X. citri* pv. *citri* strains emerged in Réunion since 2010 [23,25] but the presence of the phenotype in other SWIO islands has not been formally assessed yet. ACC was reported in the SWIO region in the 20^th^ century. ACC currently prevents the cultivation of susceptible citrus cultivars in this region, where the regular occurrence of hurricanes greatly exacerbates disease impact. Disease incidence on fruit of 30–50% (altering fruit quality and inducing early fruit drop) are commonly observed on susceptible cultivars. The earliest report was in Mauritius in 1917, and the latest was in Anjouan and Grande Comore in 2014 [26,27]. ACC was listed as present in Madagascar (EPPO global database, CABI disease map 011), but there is some doubt about disease occurrence, which has not been confirmed by laboratory analyses during grove inspections.

In the present study, we built on the high discriminatory power of microsatellite markers to assess the genetic structure of *X. citri* pv. *citri* in the SWIO region and to estimate the importance of strain circulation between the islands. A subset of strains representative of the genetic diversity was assigned to *X. citri* pv. *citri* lineages using minisatellite data. More specifically, we set out to answer the following questions: (i) Do all islands in the SWIO region host *X. citri* pv. *citri* genetic lineage 1, previously reported in Réunion, and to what extent are they related? (ii) Does this region host previously unreported lineages, and how prevalent are they? (iii) Is copper resistance present on SWIO islands other than Réunion? To answer these questions, we assembled and characterized a comprehensive strain collection, sampled from citrus in the Comoros (Anjouan, Grande Comore, Moheli and Mayotte), the Mascarenes (Mauritius, Rodrigues) and the Seychelles (Mahé). We compared them to the previously analysed *X. citri* pv. *citri* strains from Réunion [23]. Strains representative of the observed genetic diversity were further compared for pathogenicity to several *Citrus* species.

## 2. Materials and Methods

### 2.1. Bacterial Strains and Media

**Figure 1:**
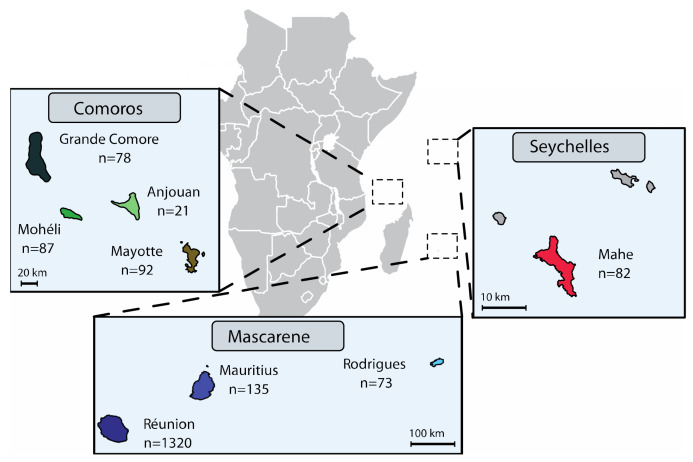
Geographical location and sample numbers of the *Xanthomonas citri* pv. *citri* strain collection analysed in the present study.

We analysed a total of 568 SWIO strains sampled from (i) the Comoros, i.e., Anjouan (n = 21), Grande Comore (n = 78), Moheli (n = 87) and Mayotte (n = 92); (ii) the Mascarenes, i.e., Mauritius (n = 135) and Rodrigues (n = 73); and (iii) the Seychelles, i.e., Mahe (n = 82) (Figure 1 and Table S1). While most strains of *X. citri* pv. *citri* were isolated in 2011–2017, the collection also included reference strains isolated between 1980 and 2010 (n = 57). These strains were compared to strains from Réunion that were analysed in a previous study [23]. Stock cultures (stored at −80°C) were initially produced from single colonies recovered after isolation from ACC symptoms, using KC semiselective medium. Subcultures of single colonies were prepared on YPGA plates [28]. Bacterial suspensions were produced in 0.01 M sterile Sigma 7–9 Tris buffer pH 7.2 (Sigma–Aldrich, Saint-Quentin Fallavier, France), unless otherwise stated. We determined copper phenotype (susceptible vs. resistant) using YPGA plates, with or without copper sulphate pentahydrate (470 mg L^−1^). Strain LH201 was used as the copper-resistant control [23].

### 2.2. Microsatellite Genotyping (MLVA-14)

The MLVA-14 scheme targeting 14 microsatellites (6–7 bp-long) in a multiplex PCR format was used to decipher the genetic structure of all SWIO *X. citri* pv. *citri* strains. We proceeded as reported earlier [17], with one exception: in the multiplex PCR assay (PCR mix pool #1), we included a primer pair targeting the *copL* gene [29], required for copper resistance of *X. citri* pv. *citri* in Réunion. Briefly, one of each primer in each PCR mix was 5′-labelled with one of the following fluorescent dyes: 6-FAM, NED, PET and VIC (Applied Biosystems, Courtaboeuf, France). As a control in each experiment, we used the *X. citri* pv. *citri* strain LN002-01, a rifamycin-resistant derivative of IAPAR 306 [30] in which the copper resistance plasmid pLH201.1 [23] was introduced by conjugation.

The number of tandem repeats at each microsatellite locus was computed from fragment length for all assayed strains using GeneMapper 4.0 (Applied Biosystems, Courtaboeuf, France) and used as input data. Nei’s unbiased estimates of genetic diversity for MLVA-14 data were calculated using the poppr 2.8.3 package [31] in R version 3.6.1 (R Core Team, 2019. A language and environment for statistical computing. R Foundation for Statistical Computing, Vienna, Austria. URL https://www.R-project.org/ (accessed on 27 April 2021)). Allelic richness (A) was calculated using the rarefaction procedure for unequal sample sizes (subsample size n = 21) using the hierfstat 0.04–22 R package [32]. Private alleles were identified using poppr. Genetic clusters (GCs) were identified as networks of haplotypes that differed by more than five microsatellite loci, using the algorithm recommended for MLVA data, combining global optimal eBURST and Euclidean distances in PHYLOViZ v1.2 [33]. Relaxed clonal complexes (RCCs) were delineated as networks of MLVA-14 haplotypes linked by up to triple-locus variations. The population structure of *X. citri* pv. *citri* was further assessed using discriminant analysis of principal components (DAPC) [34]. Here, we used the adegenet V.2.1.1. R package [35], which involved optimizing the retained number of principal components from cross-validation analysis. DAPC was used, as it is free of any assumption linked to a population genetic model (e.g., Hardy–Weinberg equilibrium or linkage equilibrium), and, thus, it is suited for analysis of datasets produced from predominantly clonal bacteria.

### 2.3. Minisatellite Typing (MLVA-31)

The MLVA-31 scheme was used to analyse the genetic relatedness (assessed from the genotyping of 31 minisatellites) between a subcollection of SWIO strains selected on the basis of MLVA-14 data (Table S1) and a worldwide strain collection of *X. citri* pv. *citri* [15,16,21] (http://bioinfo-web.mpl.ird.fr/MLVA_bank/Genotyping/view.php (accessed on 27 April 2021)). Primer pairs targeting single-locus alleles were used in a multiplex PCR format (Clontech Terra PCR Direct Polymerase Mix). One of each primer in the PCR mix was 5′-labelled with one of the following fluorescent dyes: 6-FAM, NED, PET and VIC (Applied Biosystems). PCRs and capillary electrophoresis were performed with an initial injection of 23 s, as described earlier [17]. *X. citri* pv. *citri* strain IAPAR 306 [30] was used as a control in each experiment.

Input data were obtained as described above (Section 2.2). The MLVA-31 dataset generated in this study was deposited in the *Xanthomonas citri* genotyping database (http://bioinfo-web.mpl.ird.fr/MLVA_bank/Genotyping/view.php (accessed on 27 April 2021)) [15]. PHYLOViZ v1.2 was used to build a minimum-spanning tree (MST) from the MLVA-31 dataset [33]. We assigned SWIO strains to genetic lineages using discriminant analysis of principal components (DAPC), as previously reported [16,34]. Subclusters were identified as groups of strains linked by up to triple-locus variations using PHYLOViZ [15].

### 2.4. Typeability of SWIO Strains with XAC1051-qPCR

A subset of 42 strains (Table S1), including strains assigned to all lineage 1 GCs, as well as the four pathotype A* strains, were assayed using the *X. citri* pv. *citri*-specific real-time quantitative assay XAC1051-qPCR [36]. Amplifications were performed with the Quantstudio 5 (QS5) (Applied Biosystems) real-time PCR system, using the GoTaq^®^ probe qPCR master mix kit (Promega, Charbonnières-les-Bains, France). Amplifications were carried out on 2 µL bacterial suspensions containing approximately 1 × 10^8^ cells mL^−1^ (prepared as described above) using only the primers/MGB Taqman^®^ probe specific to *X. citri* pv. *citri* (the amplification of the plant internal control was not relevant here).

### 2.5. Detached Leaf Assay-Based Pathogenicity Tests

Pathogenicity tests were conducted on eighty-six SWIO strains (all strains assigned to pathotype A* and a subcollection of lineage 1 strains, including all geographical origins selected on the basis of MLVA-14 data) (Table S1). Inoculations were performed on at least two *Citrus* species, namely, *C.* × *aurantifolia* (Mexican lime) plus one or more of the following: *C.* x *sinensis* (Washington and/or New Hall navel sweet orange), *C.* × *sinensis* × *C. reticulata* (Ortanique tangor) and *C.* x *paradisi* (Henderson, Marsh and/or Star Ruby grapefruit). We followed a method described previously [11]; the only difference here was that we used 5 µl droplets of bacterial suspensions containing approximately 1 x 10^8^ cells mL^−1^ (prepared as described above) for inoculation. Sterile buffer and strain IAPAR 306 were used as negative and positive controls, respectively.

### 2.6. Attached Leaf Assay-Based Pathogenicity and in Planta Growth of Lineage 1 Strains

Assays were conducted on three different citrus lines: New Hall navel sweet orange (*C.* × *sinensis*), Zanzibar mandarin (*C. reticulata*) and Buddha’s hand citron (*C. medica*). We used spray inoculation, because it reveals subtle differences in virulence, which are not shown by infiltration inoculation [37]. We conducted assays on sixteen *X. citri* pv. *citri* strains, four of each main GC (Table S2). Six- to eight-month-old citrus plants (three plants per strain–host combination) were used for inoculations. Plants were placed in growth chambers at 28 ± 1 °C day and 26 ± 1 °C night and 80 ± 5% relative humidity with a photoperiod of 12 h. Newly expanded young flushes were spray inoculated until run-off with bacterial suspensions containing approximately 1 × 10^8^ cells mL^−1^, prepared as described above. Plants were then covered with large clear polyethylene bags overnight. All plants were incubated for 25 days. All inoculated leaves were examined (daily during the first week after inoculation and then every other day) for lesion development. The following scale was used: 0 = no lesion; 1 = one to 10 lesions; 2 = 11 to 50 lesions; 3 ≥ 50 lesions. The susceptibility of leaves (from young citrus flushes) to stomatal infections by *X. citri* pv. *citri* is clearly age-dependent. Although stomata develop during the very early stages of leaf development, stomatal pores are not open at growth stages < 50% expansion, as shown by scanning electron microscopy [9,38]. When leaves are nearing their mature size, the fully developed cuticle acts as a protective layer against stomatal infections [8]. Therefore, we expected that, after spray inoculation, all the leaves along an actively growing flush would present different susceptibility to *X. citri* pv. *citri*. We expected to see partial resistance on older leaves. When we spray-inoculated very young leaves, we expected a delayed lesion development until the stomata became susceptible. Areas under disease progression stairs (AUDPS) were computed from disease severity data using the agricolae 1.3.3 R package [39]. AUDPS were converted into a ratio by dividing by the maximal area recorded and corrected to within ]0; 1[. A mixed generalized linear model with beta distribution was built for each host species with the strain or GC as the fixed effect and the plant as random effect using glmmTMB 1.0.2.1 R package [40]. The strain effect was analysed by deviance test using car 3.0.6 R package [41], and multiple comparisons, using emmeans 1.5.3 R package. In order to limit the heterogeneity in leaf susceptibility, the amount of censored data was estimated by maximizing the log-likelihood of the beta regression model for each host species. Binary disease status datasets were also produced from disease severity scores and were analysed using survival analysis [42] with the survival 3.1.8 [43] and survminer 0.4.6 R packages. The analysis estimates the probability for a leaf to remain healthy until a given point in time. Comparisons between strains or GCs were performed using chi-square-based test statistics [42].

Twenty-five days after inoculation (DAI), we enumerated *X. citri* pv. *citri* population sizes from single canker lesions. For each strain–cultivar combination, nine single canker lesions were collected using a sterile 8 mm punch. Each lesion was placed in a sterile microtube containing 1 mL of sterile buffer and gently agitated for 15 min. (50 rpm) at room temperature, mimicking the natural release of the bacterium from canker lesions in the presence of free water. Each lesion was then transferred to a fresh tube and homogenized for 30 s in 1 mL of sterile buffer with sterile ceramic beads in a FastPrep24 device (MP Biomedicals). Appropriate dilutions of washates and grindates were plated on semiselective KC agar plates using a Spiral plater (Interscience). Plates were incubated for 3–4 days at 28 °C, and *Xanthomonas*-like colonies were enumerated, as recommended by the manufacturer. Exuded *X. citri* pv. *citri* population sizes, expressed as log(ufc.lesion^−1^) were calculated from washate enumerations. Total *X. citri* pv. *citri* population sizes were calculated from washate + grindate enumerations. For each plant species, the strain effect on bacterial population size was analysed by ANOVA with Box–Cox power data transformation (residual variance homogenisation) using car and MASS 7.3–51.4 packages [44], followed by Tukey’s multiple pairwise comparisons using the multcomp 1.4–15 R package [45].

## 3. Results

### 3.1. Genetic Diversity among Strains from Different Islands in the SWIO Region Revealed by Microsatellite Typing and Cop-PCR

We used microsatellite typing to resolve the genetic structure among *X. citri* pv. *citri* strains at small evolutionary scales [14,16,17,21,23]. We identified 390 haplotypes among the 568 assayed strains. At the island scale, allelic richness (A) ranged from 3.1 to 5.6 (Table 1). The islands that have hosted the pathogen for long periods tended to display higher A values. The highest value of private allelic richness A_P_ (i.e., alleles solely present in strains from a single island) was found in the Seychelles population. It was also high in Réunion. For other islands, A_P_ was low to null (Anjouan and Grande Comore) (Table 1). The multiplex PCRs did not reveal *copL* PCR amplification from any assayed strain, although amplification was obtained for the copper-resistant control strain from Réunion. As expected, none of the 47 subsampled strains (originating from all sampled islands) grew on media supplemented with copper, confirming their copper-susceptible phenotype.

**Figure 2:**
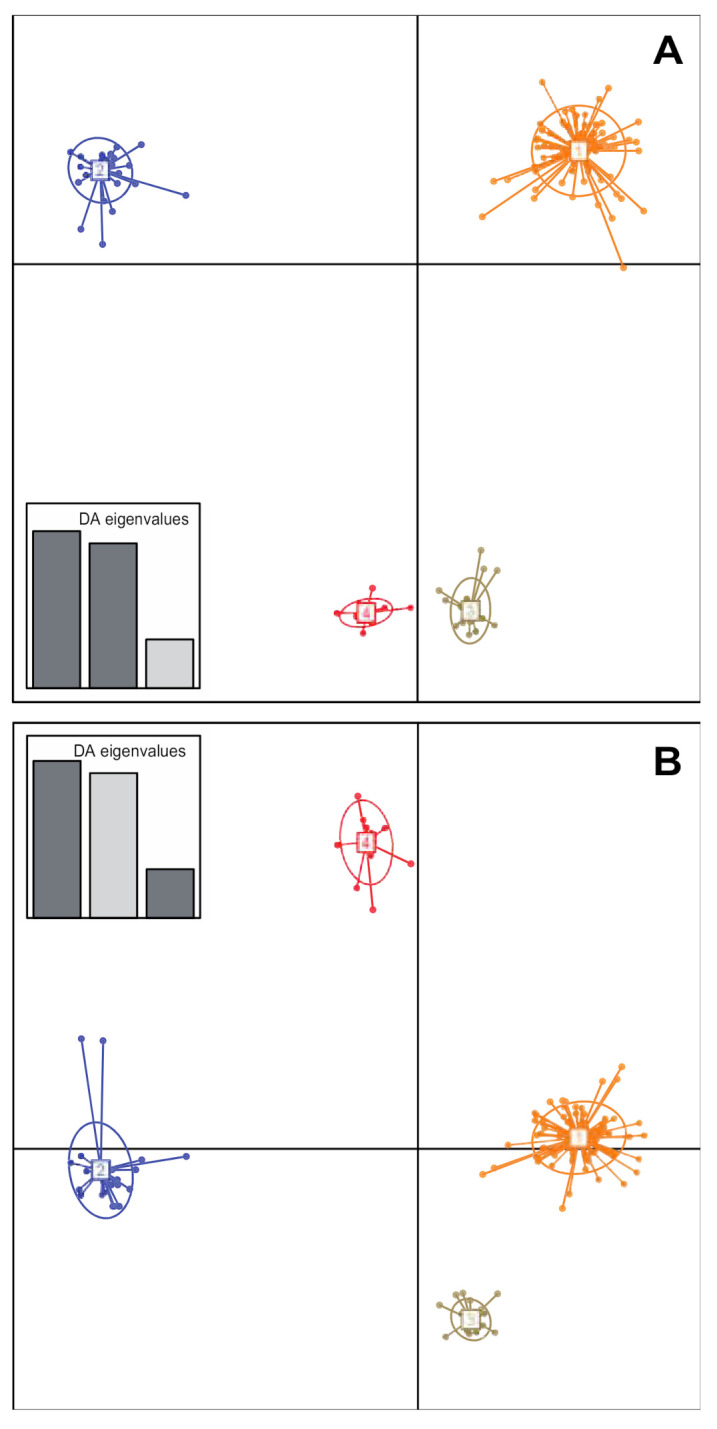
Genetic structure of *Xanthomonas citri* pv. *citri* lineage 1 in the SWIO region based on the discriminant analysis of principal components (DAPC) of microsatellite data. Numbers and colours represent the four genetic clusters retained from Bayesian information criterion (BIC) values. Strains from the Comoros and the Mascarenes grouped in a single genetic cluster (GC1), whereas strains from the Seychelles grouped in three distinct clusters. (**A**) Scatterplot representing axes 1 and 2 of the DAPC. (**B**) Scatterplot representing axes 1 and 3 of the DAPC. A subset of haplotypes originating from the Comoros and the Mascarenes (GC1, minor clusters and singletons) were used to avoid too large within-cluster variance (large haplotype number disequilibrium among clusters).

The genetic relatedness among SWIO strains was analysed from the microsatellite allelic profiles from our strain collection together with the ones previously obtained for Réunion strains [23]. The minimum spanning tree (not shown) suggested four main genetic clusters (GC) in the dataset. This structure was further supported by DAPC (Figure 2). Ninety-three percent of haplotypes identified in the Comoros (Anjouan, Grande Comore, Moheli and Mayotte) and the Mascarenes (Mauritius, Réunion and Rodrigues) clustered as GC1. The remaining haplotypes from these locations were structured as minor clusters (i.e., networks grouping ≤3 haplotypes) or singletons (i.e., haplotypes whose multilocus allelic profile differed from its closest relative at ≥6 microsatellite loci) (Table 2). Interestingly, all strains originating from the Seychelles were grouped into three distinct GCs, GC2 (n = 44), GC3 (n = 25) and GC4 (n = 13), which did not contain any haplotypes from any other island.

**Figure 3:**
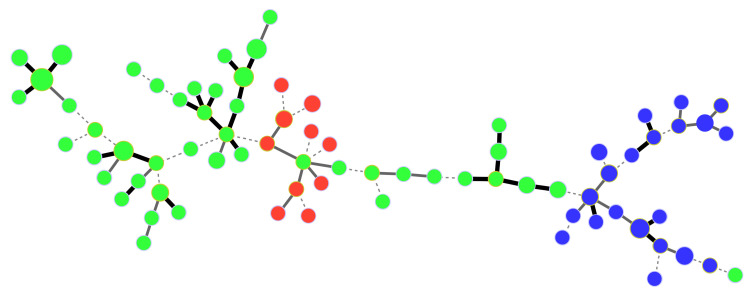
Categorical minimum spanning tree from MLVA-14 data showing the genetic relatedness among strains of *Xanthomonas citri* pv. *citri* in the Comoros. Dots represent haplotypes. Dot diameter and colour are representative of the number of strains per haplotype and island of isolation, respectively (red = Anjouan; blue = Grande Comore; green = Mayotte). Single, double and triple locus variations are represented as thick solid, thin solid and dotted lines joining haplotypes, respectively.

The structure of GC1 from the Comoros and Mascarene Archipelagos was further examined by delineating RCCs, which grouped together closely related strains, in order to identify putative epidemiological links between strains. A total of 75 RCCs, nine of which grouped ≥20 haplotypes, and 141 singletons were delineated. In the Mascarenes, very few strains originating from distinct islands grouped in the same RCC. Only two pairs of strains originating from Mauritius and Réunion (all isolated in the 1980s) shared identical haplotypes. In Réunion, RCC1, RCC2 and RCC8 included haplotypes that were distributed all over the island. In contrast, the distribution of RCC4, RCC5 and RCC7 was geographically restricted to one or two localities no more than 10 km apart. In Mauritius, most of the strains grouped in RCC6, which was distributed all over the island. In contrast, the structure of populations in Rodrigues generally had a large number of singletons and small RCCs, often restricted to a single or spatially close citrus blocks. The situation was markedly different in the Comoros, where most haplotypes originating from Grande Comore (n = 21), Anjouan (n = 10) and Mayotte (n = 46) clustered in RCC3, revealing a close genetic relatedness among strains from the three islands (Figure 3). Strains from Moheli appeared only distantly related to strains from other Comoros islands.

All 42 strains, representative of the genetic diversity among strains from different islands in the SWIO region, tested positive with the *X. citri* pv. *citri*-specific XAC1051-qPCR assay. The cycle threshold (Ct) values ranged from 10.8 to 17.5.

### 3.2. Minisatellite-Based Assignation of SWIO Strains to X. citri pv. citri Genetic Lineages

A subset of 215 strains from all GCs, minor clusters and singletons was submitted to minisatellite genotyping in order to assign them to the genetic lineages identified in *X. citri* pv. *citri* (Table 2) [15]. This complemented a dataset previously obtained from Réunion [23], yielding a total of 388 MLVA-31 genotyped SWIO strains.

Based on DAPC, most strains from the SWIO region were assigned to genetic lineage 1 (pathotype A). A network of 73 MLVA haplotypes differing by single- or double-locus variations was produced among these strains (Figure S1). Sixteen of these haplotypes were identified from at least two distinct SWIO islands. Haplotype #2, which was detected in five islands (Anjouan, Mayotte, Moheli, Mauritius and Réunion) and previously identified in different regions (Asia, Middle East, Oceania and South America) [16], was identified as the most frequent MLVA-31 haplotype in the SWIO region and as the putative founder haplotype. Although 13 minisatellite loci were shown to be polymorphic among SWIO lineage 1 strains, most (50/72) polymorphisms detected over the minimum spanning tree path occurred at three fast-evolving loci (Xcc3816, Xcc3993 and Xcc4748). Polymorphism at these three loci was previously identified among epidemiologically related strains [15]. An allelic state for locus Xcc2059 was found specific to GC2 strains (Seychelles), and the corresponding haplotypes (#168, 210 and 217) have not been identified from other countries to date [16].

Four strains isolated from Mauritius, Moheli and Réunion were assigned to genetic lineage 4, so far solely comprising pathotype A* strains (Table 2). These strains were genetically diverse (i.e., their respective allelic states differed at ≥8 minisatellite loci). The two Mauritian strains JJ238-41 and LM053-15 were single-locus variants of strains NCPPB 3615 (India) and NIGEB196-1 (Iran), respectively. Strain LK135-01 from Moheli shared the same allelic profile as the Thai strain JJ238-24, although the two strains were distinguishable using microsatellite data. The Réunion strain JN658 shared no close relatedness with other previously genotyped pathotype A* nor to any pathotype A^w^ strain.

### 3.3. Pathogenicity Assays Confirm the Prevalence of Pathotype A and the Limited Presence of Pathotype A* in the SWIO Region

A subset of strains from all GCs, minor clusters and singletons were submitted to pathogenicity tests on detached Mexican lime, as well as sweet orange, grapefruit and/or tangor leaves. Consistent with previous data, all SWIO strains that were assigned to genetic lineage 1 by minisatellite typing produced typical callus-like reactions on all assayed citrus species, similar to the positive control strain IAPAR 306. In contrast, all four strains assigned to genetic lineage 4 only produced callus-like reactions on Mexican lime, and no typical canker lesions were observed on the other citrus species assayed. This corresponds to the phenotype of the disease development index DDi III, reported earlier [46]. No lesion developed from negative controls, regardless of the citrus species assayed.

### 3.4. Relative Virulence of SWIO Strains from the Four Major Genetic Clusters on Three Citrus Species

We further assayed pathogenicity and in planta growth of selected strains from the four major GCs on attached leaves of four *Citrus* species. As expected, all 16 strains of *X. citri* pv. *citri* pathotype A lineage 1, whatever their GC assignment, started producing small canker-like lesions at 6 to 7 DAI on each assayed plant species. Based on survival analyses, the disease incidence progress of GC3 strains was greater than for the other GCs on citron and sweet orange (*p* < 0.0001). In contrast, survival analyses suggested a lack of GC-dependent response (*p* = 0.720) to inoculations on mandarin (Figures S2–S4). Differences among strains (*p*-values) are shown in Table S3.

Disease development patterns were investigated in more detail using AUDPS data. It further confirmed that GC3 was more aggressive on citron than GC1 (*p* = 0.004) and GC2 (*p* = 0.045), but not GC4 (*p* = 0.664). No significant differences among GCs were shown on mandarin and sweet orange (*p* > 0.38). The differences among strains (*p*-values) are shown in Table S4.

Large *X. citri* pv. *citri* population sizes were present in canker lesions, irrespective of the assayed bacterial strain and the citrus species at 25 DAI. Mean population sizes ranged from 4 × 10^6^ to 3 × 10^7^, 3 × 10^6^ to 4 10^7^ and 8 × 10^6^ to 4 × 10^7^ cfu lesion^−1^ on citron, mandarin and sweet orange, respectively (Table S5). When lesions were soaked in buffer, exudation of all strains was fast and massive for all assayed host species, consistent with earlier results [47,48]. Both exuded and total population sizes showed a significant strain * host interaction (ANOVA, *p* < 0.0001). Analyses were conducted again using split datasets. Differences among strains in total or exuded population sizes were ≤ 1 log unit and did not appear to be GC-related (Table 3).

## 4. Discussion

We conducted a comprehensive molecular and pathological characterization of 568 *X. citri* pv. *citri* strains from the SWIO region. The present study confirmed a high prevalence of ACC in the SWIO region (the Comoros, the Mascarenes and the Seychelles), where *X. citri* pv. *citri* causes severe repeated disease outbreaks in citrus groves. *X. citri* pv. *citri* populations in the SWIO region were genetically diverse. Importantly, no copper resistance was detected in islands other than Réunion.

### 4.1. Genetic Lineage 1 is Markedly Prevalent in the SWIO Region, Displays a Geographic Structure and Sporadically Cohabits with Nonepidemic Lineage 4 Strains

Consistent with its worldwide prevalence [15], the present study highlighted the marked predominance of pathotype A genetic lineage 1 strains in the SWIO region. Very recently, SNP data showed that genetic lineage 1 strains causing ACC in the Seychelles are genetically diverse and clearly differ from strains originating from the Comoros and the Mascarenes [49]. Although microsatellite data have recognized limitations for accurately assessing deep genetic relatedness among groups of individuals [50,51], the present study revealed large genetic differences between strains from the Seychelles vs. the Comoros and the Mascarenes. These results suggest that several distinct introduction events of *X. citri* pv. *citri* have occurred at both regional and island scale (e.g., Mahé in the Seychelles where strains isolated the same year from the same citrus species can be assigned to different GCs).

Here, we provide the first report of the presence of genetic lineage 4 pathotype A* strains in three islands in the SWIO region, namely Moheli, Mauritius and Réunion. These strains were found to be genetically diverse based on minisatellite and microsatellite data. Therefore, they are likely to represent distinct introductions in the SWIO region. The Moheli strain was distinct from but related to a Thai pathotype A* strain. The two Mauritian strains were distinct from but related to pathotype A* strains from India and Iran, respectively [21]. The pathotype A* strain from Réunion shared no close relatedness to any other pathotype A* genotyped to date. The citrus block where this strain was detected has been removed, and no other pathotype A* strains were detected despite extensive sampling [23]. Similarly, additional bacterial isolation attempts from the lime blocks in Mauritius and Moheli, where pathotype A* strains originated, confirmed the marked prevalence of pathotype A strains (data not shown). Epidemicity of pathotype A* was primarily reported from countries where lime cultivation is widespread (e.g., Iran, Ethiopia, Sudan). In the context of SWIO islands, these strains were of no agricultural significance. This is probably due to lime’s limited presence in the highly heterogeneous agricultural landscapes, which may prevent their epidemicity.

### 4.2. The Seychelles Host Genetically Diverse X. citri pv. citri Pathotype A Strains Differing in Pathogenicity

In the present study, we used a spray-inoculation technique to assess the pathogenicity of strains representative of the four GCs, which were identified in the SWIO region on three citrus species. A recent study evidenced that the structural and chemical properties of leaf surface components impact the susceptibility to *X. citri* pv. *citri*, i.e., pathogenicity differs among citrus lines. This was observed after spray inoculation but not after infiltration inoculation [52]. Interestingly, strains assigned to one of the unique groups detected in the Seychelles (GC3) differed to strains assigned to other GCs in terms of the dynamics of canker lesion development, i.e., disease incidence and AUDPS data on citron, an ancestral *Citrus* species from which some modern lines originated [7]. However, there was no marked difference in *X. citri* pv. *citri* population sizes, which were measured in canker lesions at 25 DAI, for all assayed GCs. This suggests that, compared to other GCs, GC3 strains can produce more canker lesions, which develop faster after spray inoculation, but do not survive in or exude from lesions at larger population sizes. Thus, in addition to a broad genetic polymorphism, the strains of *X. citri* pv. *citri* from the Seychelles showed greater polymorphism in terms of their pathogenicity. Given the Seychelles’ small size and relative isolation, the island of Mahé could be an interesting site for assessing gene flow patterns among admixed, genetically remote strains. This has been evidenced recently for *Xanthomonas perforans* using genomic and metagenomic approaches [53,54].

### 4.3. Microsatellite Typing Suggests that the Pathogen’s Inter- and Intra-Island Movements Differ

We found no sign of close genetic relatedness among strains originating from distinct archipelagos (i.e., no RCC grouped strains originating from distinct archipelagos). This suggests that the pathogen’s recent interisland movement through contaminated citrus material has been very limited. Consistent with this finding, no copper resistance associated with the *copLAB* system was identified in any strain originating from the SWIO islands, apart from Réunion, where it has been previously reported [23,25]. Indeed, besides a few exceptions, RCCs that were delineated among GC1 strains seemed largely island-specific.

In the Mascarenes, there was little evidence of high genetic relatedness among strains from distinct islands. The situation in the Comoros was more contrasted. Most haplotypes originating from Grande Comore, Anjouan and Mayotte displayed close genetic relatedness, suggesting that interisland dispersal of *X. citri* pv. *citri*, most likely through contaminated citrus propagative material, occurs in the Comoros. Conversely, none of the strains from Moheli appeared to be closely related to strains originating from other neighbour islands in the Comoros or, more broadly, from the SWIO region.

The intra-island distribution of haplotypes also revealed contrasting situations. Populations in Rodrigues and Moheli were structured primarily as a large number of singletons and small RCCs. The latter are frequently restricted to a single or spatially close citrus block. This structure might originate from the very low development of citrus industries. Indeed, local citrus growers often produce the plants they need for grove establishment. In addition, many strains from Rodrigues and Moheli were isolated from Mexican lime, which is generally grown for local consumption. The Mexican lime species is commonly propagated locally from seeds (which do not transmit the pathogen), thereby limiting exogenous contamination. In contrast, most of the strains from Mauritius or Réunion grouped in RCCs that were distributed all over the island, suggesting the diffusion of contaminated propagative citrus plant material from commercial nurseries. In Réunion, strains from these RCCs were also detected from commercial citrus nurseries. The trade of infected citrus nursery plants was repeatedly identified as a major pathway for *X. citri* pv. *citri* long-distance spread [14,16]. Overall, the present study emphasizes the importance of improving the control of ACC and other citrus diseases in the nurseries in the SWIO region. New legislation targeting ACC, Huanglongbing, tristeza (and their respective vectors) is currently being implemented in Réunion to improve the sanitary quality of citrus nursery plants. The XAC1051-qPCR assay, whose ability to detect all *X. citri* pv. *citri* variants identified in the SWIO region was herein confirmed, represents a valuable tool for ACC surveillance and management in the region.

## Figures and Tables

**Table 1:** Genetic diversity parameters calculated from microsatellite data (MLVA-14) for *X. citri* pv. *citri* pathotype A in the SWIO region.

Island	N ^1^	N_H_^2^	A ^3^	A_P_ ^4^	H_E_^5^	Cluster ^6^
**The Comoros**						
Anjouan	21	19	3.786	0.000	0.430	GC1 (95%)
Grande Comore	78	51	3.604	0.000	0.490	GC1 (100%)
Mayotte	92	56	3.117	0.185	0.439	GC1 (99%)
Moheli	86	78	5.644	0.082	0.666	GC1 (74%)
**The Mascarenes**						
Mauritius	133	73	4.013	0.083	0.468	GC1 (95%)
Réunion ^7^	1320	789	5.289	0.455	0.633	GC1 (98 %)
Rodrigues	73	63	5.265	0.014	0.649	GC1 (63%)
**The Seychelles**						
Mahé	82	46	3.512	0.549	0.680	GC2 (54%) GC3 (30%) GC4 (16%)

^1^ Number of isolates per island; ^2^ number of haplotypes per island; ^3^ allelic richness calculated by rarefaction method (n = 21); ^4^ private allelic richness; ^5^ Nei’s genetic diversity; ^6^ cluster number derived from analysis of microsatellite data; ^7^ data primarily from [23]. Values in brackets indicate the percentage of haplotypes assigned to GCs. Other strains were assigned to minor GCs or singletons (see Materials and Methods section for details). Strains assigned to pathotype A* (genetic lineage 4) were not considered.

**Table 2:** Genetic cluster and genetic lineage assignation of strains of *Xanthomonas citri* pv. *citri* originating from the SWIO region based on microsatellite and minisatellite genotyping data, respectively.

Cluster (Microsatellite Data)	Country (Archipelago)	Source	n	Genetic Lineage (Minisatellite Data) ^1^
GC1	Anjouan (The Comoros)	This study	16	1
GC1	Grande Comore (The Comoros)	This study	5	1
GC1	Mayotte (The Comoros)	This study	56	1
GC1	Moheli (The Comoros)	This study	22	1
GC1	Mauritius (The Mascarenes)	This study	25	1
GC1	Rodrigues (The Mascarenes)	This study	11	1
GC1	Réunion (The Mascarenes)	This study	25	1
GC1	Réunion (The Mascarenes)	[23]	162	1
GC2	Mahé (The Seychelles)	This study	13	1
GC3	Mahé (The Seychelles)	This study	6	1
GC4	Mahé (The Seychelles)	This study	5	1
minor/singleton	Anjouan (The Comoros)	This study	1	1
minor/singleton	Mayotte (The Comoros)	This study	1	1
minor/singleton	Moheli (The Comoros)	This study	18	1
minor/singleton	Moheli (The Comoros)	This study	1	4
minor/singleton	Mauritius (The Mascarenes)	This study	4	1
minor/singleton	Mauritius (The Mascarenes)	This study	2	4
minor/singleton	Rodrigues (The Mascarenes)	This study	24	1
minor/singleton	Réunion (The Mascarenes)	This study	10	1
minor/singleton	Réunion (The Mascarenes)	This study	1	4
minor/singleton	Réunion (The Mascarenes)	[23]	5	1

^1^ Assignment to genetic lineages was achieved using Discriminant Analysis of Principal Components (DAPC), as reported earlier [16], lineage 1 = pathotype A, lineage 4 = pathotype A*.

**Table 3:** Comparisons among strains of exuded or total *Xanthomonas citri* pv. *citri* population sizes enumerated from single canker lesions 25 days after spray inoculation of three host species.

		Citron		Mandarin		Sweet Orange	
		Exuded	Total	Exuded	Total	Exuded	Total
ANOVA probability		0.002	0.004	0.006	< 0.001	< 0.001	0.025
GC	Strain	Log-transformed cfu lesion^−1^ compared using Tukey’s tests					
1	LH241	6.69 ^ab^	7.43 ^a^	6.20 ^ab^	7.33 ^abc^	6.97 ^ab^	7.43 ^ab^
1	LM089-41	7.00 ^a^	7.41 ^a^	6.63 ^a^	7.62 ^a^	6.88 ^abc^	7.27 ^ab^
1	LN005-4	6.65 ^ab^	7.08 ^ab^	6.20 ^ab^	6.99 ^abc^	6.91 ^abc^	7.58 ^a^
1	LN007-3	6.47 ^b^	7.30 ^ab^	6.30 ^ab^	7.21 ^abc^	6.79 ^abc^	7.47 ^ab^
2	JZ092	6.45 ^b^	7.12 ^ab^	6.29 ^ab^	7.19 ^abc^	6.98 ^abc^	7.32 ^ab^
2	LB100-1	6.56 ^ab^	7.14 ^ab^	5.93 ^ab^	6.79 ^bc^	6.52 ^c^	6.90 ^b^
2	LJ001	6.54 ^b^	6.92 ^ab^	6.50 ^ab^	7.49 ^ab^	6.72 ^abc^	7.07 ^ab^
2	LP029-15	6.80 ^ab^	7.09 ^ab^	5.65 ^ab^	6.60 ^c^	6.39 ^c^	7.19 ^ab^
3	JZ094	6.16 ^b^	6.75 ^ab^	6.12 ^ab^	7.13 ^abc^	6.70 ^abc^	7.01 ^ab^
3	LP027-3	6.75 ^ab^	7.28 ^ab^	5.64 ^ab^	6.49 ^c^	6.88 ^abc^	7.38 ^ab^
3	LP027-5	6.32 ^b^	6.64 ^b^	6.49 ^ab^	7.07 ^abc^	6.60 ^bc^	7.23 ^ab^
3	LP027-13	6.58 ^ab^	7.06 ^ab^	5.85 ^ab^	6.89 ^abc^	6.52 ^c^	7.12 ^ab^
4	LP028-2	6.84 ^ab^	7.36 ^a^	5.60 ^b^	6.57 ^c^	6.46 ^bc^	7.17 ^ab^
4	LP028-3	6.73 ^ab^	7.15 ^ab^	5.70 ^ab^	6.64 ^c^	7.02 ^a^	7.39 ^ab^
4	LP028-5	6.66 ^ab^	7.00 ^ab^	5.92 ^ab^	6.63 ^bc^	6.63 ^abc^	7.34 ^ab^
4	LP028-6	6.80 ^ab^	7.16 ^ab^	6.11 ^ab^	6.82 ^c^	6.69 ^abc^	7.31 ^ab^

Population sizes were determined on KC semiselective medium. Nine lesions per strain–host combination were used for assessing population size. Values (i.e., means) followed by the same letter(s) are not significantly different (*p* = 0.05) based on Tukey’s tests.

## Data Availability

All data produced in this study are presented in this publication. Minisatellite data are available in the *Xanthomonas citri* genotyping database (http://bioinfo-web.mpl.ird.fr/MLVA_bank/Genotyping/view.php (accessed on 27 April 2021)).

## Supplementary Materials

The following are available online at https://www.mdpi.com/article/10.3390/microorganisms9050945/s1, Figure S1: Minimum spanning tree representing the genetic relatedness based on minisatellite genotyping (MLVA-31) among lineage 1 strains of *Xanthomonas citri* pv. *citri* from the SWIO region. Dot diameter is representative of the number of strains per haplotype. Dot colour is representative of the archipelago/island of isolation (Comoros Archipelago: light green for Anjouan; medium green for Moheli; dark green for Grande Comore; khaki for Mayotte. Mascarene Archipelago: light blue for Rodrigues; medium blue for Mauritius; dark blue for Réunion. Seychelles Archipelago: red for Mahé). Single and double locus variations are represented as thick and thin solid lines joining haplotypes, respectively, Figure S2: Survival probability of citron (*Citrus medica*) leaf infection by *Xanthomonas citri* pv. *citri* over time, Figure S3: Survival probability of sweet orange (*Citrus* x *sinensis*) leaf infection by *Xanthomonas citri* pv. *citri* over time, Figure S4: Survival probability of mandarin (*Citrus reticulata*) leaf infection by *Xanthomonas citri* pv. *citri* over time, Table S1: *Xanthomonas citri* pv. *citri* strains isolated from citrus in the South West Indian Ocean (SWIO) region, Table S2: *Xanthomonas citri* pv. *citri* lineage 1 strains used for spray-inoculation experiments of three citrus species, Table S3: Pairwise strain comparisons (*p*-values) based on disease incidence data on citron, mandarin and sweet orange, Table S4 Pairwise strain comparisons (*p*-values) based on AUDPS data on citron, mandarin and sweet orange, Table S5: Comparisons among citrus species of exuded and total *Xanthomonas citri* pv. *citri* population sizes recorded from single canker lesions 25 days after spray-inoculation of 16 *X. citri* pv. *citri* strains.
