# Supplementary material for: Diversity and Geographical Structure of Xanthomonas citri pv. citri on Citrus in the South West Indian Ocean Region
Source: Microorganisms. 2021 Apr 27;9(5):945. doi: 10.3390/microorganisms9050945 (PMC8146439; doi:10.3390/microorganisms9050945)
Supplement: Supplementary file 1 [file microorganisms-09-00945-s001.zip › V2/Table S5-2.docx]

**Table S5** Exuded and total *Xanthomonas citri* pv. *citri* population sizes enumerated from single canker lesions 25 days after spray-inoculation for three citrus species inoculated with 16 *X. citri* pv.  *citri* strains

|  |  | GC1 | | | | GC2 | | | | GC3 | | | | GC4 | | | |
| --- | --- | --- | --- | --- | --- | --- | --- | --- | --- | --- | --- | --- | --- | --- | --- | --- | --- |
| Host | Type | LH241 | LM089-41 | LN005-4 | LN007-3 | JZ092 | LB100-1 | LJ001 | LP029-15 | JZ094 | LP027-3 | LP027-5 | LP027-13 | LP028-2 | LP028-3 | LP028-5 | LP028-6 |
| Citron | Exuded | 6.69 ab | 7.00 a | 6.65 a | 6.47 a | 6.45 b | 6.56 ab | 6.54 a | 6.80 a | 6.16 a | 6.75 a | 6.32 a | 6.58 ab | 6.84 a | 6.73 ab | 6.66 a | 6.80 a |
| Mandarin | Exuded | 6.20 b | 6.63 b | 6.20 b | 6.30 a | 6.29 b | 5.93 b | 6.50 a | 5.65 b | 6.12 a | 5.64 b | 6.49 a | 5.85 b | 5.60 b | 5.70 c | 5.92 b | 6.11 b |
| Orange | Exuded | 6.97 a | 6.88 ab | 6.91 a | 6.79 a | 6.98 a | 6.52 ab | 6.72 a | 6.39 a | 6.70 a | 6.88 a | 6.60 a | 6.52 ab | 6.46 ab | 7.02 a | 6.63 a | 6.69 a |
| Citron | Total | 7.43 a | 7.41 ab | 7.08 b | 7.30 a | 7.12 a | 7.14 a | 6.92 c | 7.09 a | 6.75 a | 7.28 a | 6.64 b | 7.06 a | 7.36 a | 7.15 ab | 7.00 a | 7.16 a |
| Mandarin | Total | 7.33 a | 7.62 a | 6.99 b | 7.21 a | 7.19 a | 6.79 a | 7.49 a | 6.60 b | 7.13 a | 6.49 b | 7.07 ab | 6.89 a | 6.57 b | 6.64 b | 6.63 a | 6.82 a |
| Orange | Total | 7.43 a | 7.27 b | 7.58 a | 7.47 a | 7.32 a | 6.90 a | 7.07 bc | 7.19 ab | 7.01 a | 7.38 a | 7.23 a | 7.12 a | 7.17 ab | 7.39 a | 7.34 a | 7.31 a |

Population sizes were determined on KC semiselective medium. Nine lesions per strain–host combination were used for population size assessment. Values (*i.e.*, means) followed by the same letter(s) are not significantly different (p >= 0.05) based on Tukey’s tests.
