# Supplementary material for: Diversity and Geographical Structure of Xanthomonas citri pv. citri on Citrus in the South West Indian Ocean Region
Source: Microorganisms. 2021 Apr 27;9(5):945. doi: 10.3390/microorganisms9050945 (PMC8146439; doi:10.3390/microorganisms9050945)
Supplement: Supplementary file 1 [file microorganisms-09-00945-s001.zip › V2/Table S2.docx]

**Table S2** *Xanthomonas citri* pv. *citri* lineage 1 strains used for spray-inoculation experiments of three citrus species.

| Strain number | Host of isolation | Origin | Year | Site | GC^b^ |
| --- | --- | --- | --- | --- | --- |
| LH241 | *C. hystrix* | Réunion | 2010 | Saint Pierre | 1 |
| LM089-41 | *C. reticulata X C. x sinensis* | Réunion | 2015 | Piton Hyacinthe | 1 |
| LN005-4 | *C. hystrix* | Réunion | 2016 | Saint Pierre | 1 |
| LN007-3 | *C. x limon* | Réunion | 2016 | Piton Hyacinthe | 1 |
| JZ092 | *C. x limon* | Seychelles | 2003 | NA^a^ | 2 |
| LB100-1 | *C. x sinensis X P. trifoliata* | Seychelles | 2005 | Grand Anse | 2 |
| LJ001 | *Citrus*sp. | Seychelles | 2012 | La Passe | 2 |
| LP029-15 | *C. x sinensis* | Seychelles | 2017 | Anse Boileau | 2 |
| JZ094 | *C. x limon* | Seychelles | 2003 | Anse Royale | 3 |
| LP027-3 | *C. x aurantiifolia* | Seychelles | 2017 | Le Cap | 3 |
| LP027-5 | *C. x aurantiifolia* | Seychelles | 2017 | Le Cap | 3 |
| LP027-13 | *C. x aurantiifolia* | Seychelles | 2017 | Le Cap | 3 |
| LP028-2 | *C. x aurantiifolia* | Seychelles | 2017 | Baie Lazare | 4 |
| LP028-3 | *C. x aurantiifolia* | Seychelles | 2017 | Baie Lazare | 4 |
| LP028-5 | *C. x aurantiifolia* | Seychelles | 2017 | Baie Lazare | 4 |
| LP028-6 | *C. x aurantiifolia* | Seychelles | 2017 | Baie Lazare | 4 |

^a^ NA: not available.

^b^Genetic clusters as determined by microsatellite typing.
