# Supplementary material for: Diversity and Geographical Structure of Xanthomonas citri pv. citri on Citrus in the South West Indian Ocean Region
Source: Microorganisms. 2021 Apr 27;9(5):945. doi: 10.3390/microorganisms9050945 (PMC8146439; doi:10.3390/microorganisms9050945)
Supplement: Supplementary file 1 [file microorganisms-09-00945-s001.zip › V2/Table S1.docx]

**Table S1.** *Xanthomonas citri* pv*. citri* strains isolated from *Citrus* in the South West Indian Ocean (SWIO) region.

| Strain number | Host of isolation | Archipelago | Island | Year | MLVA-14 haplotype number | MLVA-14 cluster^a^ | MLVA-31 haplotype number | Lineage^b^ |
| --- | --- | --- | --- | --- | --- | --- | --- | --- |
| LK126-01 ^c^ | Mexican lime | Comoros | Anjouan | 2013 | 2571 | 1 | 35 | 1 |
| LK126-02 | Mexican lime | Comoros | Anjouan | 2013 | 2572 | 1 | 2 | 1 |
| LK126-03 | Mexican lime | Comoros | Anjouan | 2013 | 2728 | 1 | 2 | 1 |
| LK126-04 | Mexican lime | Comoros | Anjouan | 2013 | 2573 | 1 | NA |  |
| LK126-07 | Mexican lime | Comoros | Anjouan | 2013 | 2574 | 1 | 2 | 1 |
| LK126-08 | Mexican lime | Comoros | Anjouan | 2013 | 2575 | 1 | 2 | 1 |
| LK127-01 | Sweet orange | Comoros | Anjouan | 2013 | 2576 | 1 | NA |  |
| LK127-02 | Sweet orange | Comoros | Anjouan | 2013 | 2577 | 1 | 100 | 1 |
| LK127-03 | Sweet orange | Comoros | Anjouan | 2013 | 2578 | 1 | NA |  |
| LK127-05 | Sweet orange | Comoros | Anjouan | 2013 | 2579 | 1 | 186 | 1 |
| LK127-06 | Sweet orange | Comoros | Anjouan | 2013 | 2580 | 1 | 100 | 1 |
| LK127-07 | Sweet orange | Comoros | Anjouan | 2013 | 2581 | 1 | 100 | 1 |
| LK127-08 | Sweet orange | Comoros | Anjouan | 2013 | 2579 | 1 | 186 | 1 |
| LK128-01 ^c^ | Sweet orange | Comoros | Anjouan | 2013 | 2582 | 1 | 2 | 1 |
| LK128-02 | Sweet orange | Comoros | Anjouan | 2013 | 2583 | 1 | 2 | 1 |
| LK129-01 | Mexican lime | Comoros | Anjouan | 2013 | 2584 | 1 | 2 | 1 |
| LK129-02 | Mexican lime | Comoros | Anjouan | 2013 | 2585 | 1 | 11 | 1 |
| LK129-03 | Mexican lime | Comoros | Anjouan | 2013 | 2586 | 1 | 2 | 1 |
| LK129-04 | Mexican lime | Comoros | Anjouan | 2013 | 2587 |  | NA |  |
| LK129-05 | Mexican lime | Comoros | Anjouan | 2013 | 2585 | 1 | 11 | 1 |
| LK129-06 | Mexican lime | Comoros | Anjouan | 2013 | 2588 | 1 | 2 | 1 |
| JN564 | *Citru*s sp. | Comoros | Grande Comore | 1993 | 2342 | 1 | 184 | 1 |
| LA087-01 | *Citru*s sp. | Comoros | Grande Comore | 2004 | 2490 | 1 | 35 | 1 |
| LA087-02 ^c^ | *Citru*s sp. | Comoros | Grande Comore | 2004 | 2491 | 1 | 35 | 1 |
| LK144-01 | *Citru*s sp. | Comoros | Grande Comore | 2013 | 2668 | 1 | NA |  |
| LK144-02 | *Citru*s sp. | Comoros | Grande Comore | 2013 | 2669 | 1 | NA |  |
| LK144-03 | *Citru*s sp. | Comoros | Grande Comore | 2013 | 2669 | 1 | NA |  |
| LK144-04 | *Citru*s sp. | Comoros | Grande Comore | 2013 | 2668 | 1 | NA |  |
| LK144-05 | *Citru*s sp. | Comoros | Grande Comore | 2013 | 2670 | 1 | NA |  |
| LK144-06 | *Citru*s sp. | Comoros | Grande Comore | 2013 | 2671 | 1 | NA |  |
| LK144-07 | *Citru*s sp. | Comoros | Grande Comore | 2013 | 2672 | 1 | NA |  |
| LK144-08 ^c^ | *Citru*s sp. | Comoros | Grande Comore | 2013 | 2673 | 1 | NA |  |
| LK144-09 | *Citru*s sp. | Comoros | Grande Comore | 2013 | 2674 | 1 | NA |  |
| LK144-10 | *Citru*s sp. | Comoros | Grande Comore | 2013 | 2675 | 1 | NA |  |
| LK145-01 | *Citru*s sp. | Comoros | Grande Comore | 2013 | 2675 | 1 | NA |  |
| LK145-02 | *Citru*s sp. | Comoros | Grande Comore | 2013 | 2671 | 1 | NA |  |
| LK145-03 | *Citru*s sp. | Comoros | Grande Comore | 2013 | 2676 | 1 | NA |  |
| LK145-04 | *Citru*s sp. | Comoros | Grande Comore | 2013 | 2669 | 1 | NA |  |
| LK145-05 | *Citru*s sp. | Comoros | Grande Comore | 2013 | 2677 | 1 | 184 | 1 |
| LK145-06 | *Citru*s sp. | Comoros | Grande Comore | 2013 | 2678 | 1 | NA |  |
| LK145-07 | *Citru*s sp. | Comoros | Grande Comore | 2013 | 2679 | 1 | NA |  |
| LK145-08 | *Citru*s sp. | Comoros | Grande Comore | 2013 | 2680 | 1 | NA |  |
| LK145-09 | *Citru*s sp. | Comoros | Grande Comore | 2013 | 2681 | 1 | NA |  |
| LK145-10 | *Citru*s sp. | Comoros | Grande Comore | 2013 | 2678 | 1 | 195 | 1 |
| LK145-11 | *Citru*s sp. | Comoros | Grande Comore | 2013 | 2682 | 1 | NA |  |
| LK145-12 | *Citru*s sp. | Comoros | Grande Comore | 2013 | 2669 | 1 | NA |  |
| LK145-13 | *Citru*s sp. | Comoros | Grande Comore | 2013 | 2678 | 1 | 195 | 1 |
| LK145-14 | *Citru*s sp. | Comoros | Grande Comore | 2013 | 2683 | 1 | NA |  |
| LK145-15 | *Citru*s sp. | Comoros | Grande Comore | 2013 | 2684 | 1 | NA |  |
| LK145-16 | *Citru*s sp. | Comoros | Grande Comore | 2013 | 2675 | 1 | NA |  |
| LK146-01 | *Citru*s sp. | Comoros | Grande Comore | 2013 | 2685 | 1 | NA |  |
| LK146-02 | *Citru*s sp. | Comoros | Grande Comore | 2013 | 2686 | 1 | NA |  |
| LK147-01 | *Citru*s sp. | Comoros | Grande Comore | 2013 | 2687 | 1 | NA |  |
| LK147-03 | *Citru*s sp. | Comoros | Grande Comore | 2013 | 2688 | 1 | NA |  |
| LK147-04 | *Citru*s sp. | Comoros | Grande Comore | 2013 | 2689 | 1 | 196 | 1 |
| LK147-05 | *Citru*s sp. | Comoros | Grande Comore | 2013 | 2686 | 1 | 195 | 1 |
| LK147-06 | *Citru*s sp. | Comoros | Grande Comore | 2013 | 2671 | 1 | NA |  |
| LK148-01 | *Citru*s sp. | Comoros | Grande Comore | 2013 | 2690 | 1 | NA |  |
| LK148-02 | *Citru*s sp. | Comoros | Grande Comore | 2013 | 2690 | 1 | NA |  |
| LK148-03 | *Citru*s sp. | Comoros | Grande Comore | 2013 | 2691 | 1 | NA |  |
| LK149-01 | *Citru*s sp. | Comoros | Grande Comore | 2013 | 2671 | 1 | NA |  |
| LK149-02 | *Citru*s sp. | Comoros | Grande Comore | 2013 | 2692 | 1 | NA |  |
| LK149-03 | *Citru*s sp. | Comoros | Grande Comore | 2013 | 2677 | 1 | NA |  |
| LK149-04 | *Citru*s sp. | Comoros | Grande Comore | 2013 | 2671 | 1 | NA |  |
| LK149-05 | *Citru*s sp. | Comoros | Grande Comore | 2013 | 2671 | 1 | NA |  |
| LK149-06 | *Citru*s sp. | Comoros | Grande Comore | 2013 | 2677 | 1 | NA |  |
| LK149-07 | *Citru*s sp. | Comoros | Grande Comore | 2013 | 2693 | 1 | NA |  |
| LK150-01 | *Citru*s sp. | Comoros | Grande Comore | 2013 | 2694 | 1 | NA |  |
| LL051-01 | Sweet orange | Comoros | Grande Comore | 2014 | 2695 | 1 | NA |  |
| LL051-02 | Sweet orange | Comoros | Grande Comore | 2014 | 2695 | 1 | NA |  |
| LL051-03 | Sweet orange | Comoros | Grande Comore | 2014 | 2696 | 1 | NA |  |
| LL052-01 | Sweet orange | Comoros | Grande Comore | 2014 | 2697 | 1 | NA |  |
| LL053-01 | Sweet orange | Comoros | Grande Comore | 2014 | 2698 | 1 | NA |  |
| LL053-02 | Sweet orange | Comoros | Grande Comore | 2014 | 2699 | 1 | NA |  |
| LL053-03 | Sweet orange | Comoros | Grande Comore | 2014 | 2700 | 1 | NA |  |
| LL053-04 | Sweet orange | Comoros | Grande Comore | 2014 | 2701 | 1 | NA |  |
| LL053-05 | Sweet orange | Comoros | Grande Comore | 2014 | 2701 | 1 | NA |  |
| LL053-06 | Sweet orange | Comoros | Grande Comore | 2014 | 2701 | 1 | NA |  |
| LL053-07 | Sweet orange | Comoros | Grande Comore | 2014 | 2698 | 1 | NA |  |
| LL054-01 | Sweet orange | Comoros | Grande Comore | 2014 | 2702 | 1 | NA |  |
| LL054-02 | Sweet orange | Comoros | Grande Comore | 2014 | 2702 | 1 | NA |  |
| LL055-01 | Sweet orange | Comoros | Grande Comore | 2014 | 2704 | 1 | NA |  |
| LL055-02 | Sweet orange | Comoros | Grande Comore | 2014 | 2700 | 1 | NA |  |
| LL055-03 | Sweet orange | Comoros | Grande Comore | 2014 | 2700 | 1 | NA |  |
| LL055-04 | Sweet orange | Comoros | Grande Comore | 2014 | 2700 | 1 | NA |  |
| LL068-01 | *Citru*s sp. | Comoros | Grande Comore | 2014 | 2705 | 1 | NA |  |
| LL068-02 | *Citru*s sp. | Comoros | Grande Comore | 2014 | 2706 | 1 | NA |  |
| LL068-03 | *Citru*s sp. | Comoros | Grande Comore | 2014 | 2707 | 1 | NA |  |
| LL068-04 | *Citru*s sp. | Comoros | Grande Comore | 2014 | 2708 | 1 | NA |  |
| LL068-05 | *Citru*s sp. | Comoros | Grande Comore | 2014 | 2709 | 1 | NA |  |
| LL068-06 | *Citru*s sp. | Comoros | Grande Comore | 2014 | 2710 | 1 | NA |  |
| LL068-07 | *Citru*s sp. | Comoros | Grande Comore | 2014 | 2711 | 1 | NA |  |
| LL068-08 | *Citru*s sp. | Comoros | Grande Comore | 2014 | 2712 | 1 | NA |  |
| LL068-09 | *Citru*s sp. | Comoros | Grande Comore | 2014 | 2710 | 1 | NA |  |
| LL068-10 | *Citru*s sp. | Comoros | Grande Comore | 2014 | 2713 | 1 | NA |  |
| LL068-11 | *Citru*s sp. | Comoros | Grande Comore | 2014 | 2714 | 1 | NA |  |
| LL068-12 | *Citru*s sp. | Comoros | Grande Comore | 2014 | 2715 | 1 | NA |  |
| LL068-13 | *Citru*s sp. | Comoros | Grande Comore | 2014 | 2716 | 1 | NA |  |
| LL068-14 | *Citru*s sp. | Comoros | Grande Comore | 2014 | 2710 | 1 | NA |  |
| LK130-03 | Mexican lime | Comoros | Moheli | 2013 | 2589 | 1 | NA |  |
| LK130-04 ^c^ | Mexican lime | Comoros | Moheli | 2013 | 2590 | 1 | NA |  |
| LK130-05 | Mexican lime | Comoros | Moheli | 2013 | 2591 | 1 | NA |  |
| LK130-06 | Mexican lime | Comoros | Moheli | 2013 | 2592 | m17 | 2 | 1 |
| LK130-07 | Mexican lime | Comoros | Moheli | 2013 | 2593 | m17 | NA |  |
| LK130-08 | Mexican lime | Comoros | Moheli | 2013 | 2594 | 1 | 51 | 1 |
| LK130-09 | Mexican lime | Comoros | Moheli | 2013 | 2595 | m6 | 4 | 1 |
| LK130-10 | Mexican lime | Comoros | Moheli | 2013 | 2596 | m6 | NA |  |
| LK130-11 | Mexican lime | Comoros | Moheli | 2013 | 2597 | sing. | 187 | 1 |
| LK130-12 | Mexican lime | Comoros | Moheli | 2013 | 2598 | m6 | NA |  |
| LK130-13 | Mexican lime | Comoros | Moheli | 2013 | 2599 | 1 | NA |  |
| LK130-14 | Mexican lime | Comoros | Moheli | 2013 | 2600 | 1 | NA |  |
| LK131-01 | Sweet orange | Comoros | Moheli | 2013 | 2601 | 1 | 4 | 1 |
| LK131-02 | Sweet orange | Comoros | Moheli | 2013 | 2602 | 1 | NA |  |
| LK131-03 | Sweet orange | Comoros | Moheli | 2013 | 2603 | 1 | NA |  |
| LK131-04 | Mexican lime | Comoros | Moheli | 2013 | 2604 | 1 | 173 | 1 |
| LK131-05 | Mexican lime | Comoros | Moheli | 2013 | 2605 | 1 | NA |  |
| LK131-06 | Mexican lime | Comoros | Moheli | 2013 | 2606 | 1 | NA |  |
| LK131-07 | Mexican lime | Comoros | Moheli | 2013 | 2607 | 1 | 35 | 1 |
| LK131-08 | Mexican lime | Comoros | Moheli | 2013 | 2608 | 1 | NA |  |
| LK131-09 | Mexican lime | Comoros | Moheli | 2013 | 2609 | 1 | NA |  |
| LK131-10 | Mexican lime | Comoros | Moheli | 2013 | 2610 | m19 | 197 | 1 |
| LK132-01 | Mexican lime | Comoros | Moheli | 2013 | 2611 | 1 | NA |  |
| LK132-02 | Mexican lime | Comoros | Moheli | 2013 | 2612 | 1 | 189 | 1 |
| LK132-03 ^c^ | Sweet orange | Comoros | Moheli | 2013 | 2613 | 1 | 190 | 1 |
| LK132-04 | Sweet orange | Comoros | Moheli | 2013 | 2614 | 1 | NA |  |
| LK132-05 | Lemon | Comoros | Moheli | 2013 | 2615 | 1 | 191 | 1 |
| LK132-06 | Mexican lime | Comoros | Moheli | 2013 | 2616 | 1 | NA |  |
| LK132-07 | Mexican lime | Comoros | Moheli | 2013 | 2617 | 1 | NA |  |
| LK132-08 | Mexican lime | Comoros | Moheli | 2013 | 2618 | 1 | 192 | 1 |
| LK132-09 | Mexican lime | Comoros | Moheli | 2013 | 2619 | 1 | 193 | 1 |
| LK132-10 | Mexican lime | Comoros | Moheli | 2013 | 2620 | 1 | 188 | 1 |
| LK132-11 | Sweet orange | Comoros | Moheli | 2013 | 2621 | 1 | NA |  |
| LK132-12 | Sweet orange | Comoros | Moheli | 2013 | 2622 | 1 | NA |  |
| **LK135-01 ^c^** | ***Citru*s sp.** | **Comoros** | **Moheli** | **2013** | **AS0591** | **sing.** | **18** | **4** |
| LK135-02 | *Citru*s sp. | Comoros | Moheli | 2013 | 2624 | m15 | 12 | 1 |
| LK135-03 | *Citru*s sp. | Comoros | Moheli | 2013 | 2625 | m15 | 12 | 1 |
| LK136-01 | Mexican lime | Comoros | Moheli | 2013 | 2626 | sing. | 4 | 1 |
| LK136-02 | Mexican lime | Comoros | Moheli | 2013 | 2607 | m11 | 2 | 1 |
| LK136-03 | Mexican lime | Comoros | Moheli | 2013 | 2627 | m11 | 2 | 1 |
| LK136-04 | Mexican lime | Comoros | Moheli | 2013 | 2628 | m11 | 35 | 1 |
| LK136-05 | Mexican lime | Comoros | Moheli | 2013 | 2629 | sing. | 2 | 1 |
| LK136-07 | Mexican lime | Comoros | Moheli | 2013 | 2627 | m11 | NA |  |
| LK136-08 | Mexican lime | Comoros | Moheli | 2013 | 2631 | m6 | 4 | 1 |
| LK136-09 | Mexican lime | Comoros | Moheli | 2013 | 2632 | m6 | NA |  |
| LK137-01 | Sweet orange | Comoros | Moheli | 2013 | 2633 | sing. | 186 | 1 |
| LK137-02 | Mexican lime | Comoros | Moheli | 2013 | 2634 | sing. | 141 | 1 |
| LK141-01 | Sweet orange | Comoros | Moheli | 2013 | 2635 | m14 | 194 | 1 |
| LK141-02 | Sweet orange | Comoros | Moheli | 2013 | 2636 | 1 | NA |  |
| LK141-03 | Sweet orange | Comoros | Moheli | 2013 | 2637 | m14 | 194 | 1 |
| LK141-04 | Sweet orange | Comoros | Moheli | 2013 | 2637 | 1 | 194 | 1 |
| LK141-05 | Sweet orange | Comoros | Moheli | 2013 | 2638 | 1 | NA |  |
| LK141-06 | Sweet orange | Comoros | Moheli | 2013 | 2639 | 1 | 2 | 1 |
| LK141-07 | Sweet orange | Comoros | Moheli | 2013 | 2640 | 1 | NA |  |
| LK141-08 | Sweet orange | Comoros | Moheli | 2013 | 2641 | 1 | NA |  |
| LK141-09 | Sweet orange | Comoros | Moheli | 2013 | 2642 | 1 | NA |  |
| LK141-11 | Sweet orange | Comoros | Moheli | 2013 | 2643 | 1 | 2 | 1 |
| LK141-12 | Sweet orange | Comoros | Moheli | 2013 | 2644 | 1 | 2 | 1 |
| LK141-13 | Sweet orange | Comoros | Moheli | 2013 | 2645 | 1 | 2 | 1 |
| LK141-14 | Sweet orange | Comoros | Moheli | 2013 | 2645 | 1 | 2 | 1 |
| LK141-15 | Sweet orange | Comoros | Moheli | 2013 | 2646 | 1 | 2 | 1 |
| LK141-17 | Sweet orange | Comoros | Moheli | 2013 | 2647 | 1 | 2 | 1 |
| LK141-18 | Sweet orange | Comoros | Moheli | 2013 | 2644 | 1 | 2 | 1 |
| LK142-01 | Mexican lime | Comoros | Moheli | 2013 | 2648 | 1 | 2 | 1 |
| LK142-03 | Mexican lime | Comoros | Moheli | 2013 | 2649 | 1 | NA |  |
| LK142-04 | Mexican lime | Comoros | Moheli | 2013 | 2650 | 1 | NA |  |
| LK142-05 | Mexican lime | Comoros | Moheli | 2013 | 2651 | 1 | 2 | 1 |
| LK143-01 | Sweet orange | Comoros | Moheli | 2013 | 2652 | 1 | NA |  |
| LK143-02 | Mexican lime | Comoros | Moheli | 2013 | 2653 | 1 | 11 | 1 |
| LK143-03 | Mexican lime | Comoros | Moheli | 2013 | 2654 | 1 | NA |  |
| LK143-04 | Mexican lime | Comoros | Moheli | 2013 | 2655 | 1 | NA |  |
| LK143-05 | Mexican lime | Comoros | Moheli | 2013 | 2656 | 1 | NA |  |
| LK143-06 | Mexican lime | Comoros | Moheli | 2013 | 2657 | 1 | NA |  |
| LK143-07 | Mexican lime | Comoros | Moheli | 2013 | 2658 | 1 | 2 | 1 |
| LK143-08 | Mexican lime | Comoros | Moheli | 2013 | 2659 | 1 | NA |  |
| LK143-09 | Mexican lime | Comoros | Moheli | 2013 | 2660 | 1 | NA |  |
| LK143-10 | Mexican lime | Comoros | Moheli | 2013 | 2661 | 1 | NA |  |
| LK143-11 | Mexican lime | Comoros | Moheli | 2013 | 2662 | 1 | NA |  |
| LK143-12 | Mexican lime | Comoros | Moheli | 2013 | 2663 | 1 | NA |  |
| LK143-13 | Mexican lime | Comoros | Moheli | 2013 | 2664 | 1 | NA |  |
| LK143-14 | Mexican lime | Comoros | Moheli | 2013 | 2660 | 1 | NA |  |
| LK143-15 | Mexican lime | Comoros | Moheli | 2013 | 2660 | 1 | NA |  |
| LK143-16 | Mexican lime | Comoros | Moheli | 2013 | 2660 | 1 | NA |  |
| LK143-17 | Mexican lime | Comoros | Moheli | 2013 | 2665 | 1 | NA |  |
| LK143-18 | Mexican lime | Comoros | Moheli | 2013 | 2666 | 1 | 2 | 1 |
| LK143-19 | Mexican lime | Comoros | Moheli | 2013 | 2667 | 1 | NA |  |
| LK143-20 | Mexican lime | Comoros | Moheli | 2013 | 2652 | 1 | 2 | 1 |
| LJ123-01 | Sweet orange | Comoros | Mayotte | 2012 | 2515 | 1 | NA |  |
| LJ123-02 | Sweet orange | Comoros | Mayotte | 2012 | 2516 | 1 | NA |  |
| LJ124-01 | Sweet orange | Comoros | Mayotte | 2012 | 2517 | 1 | NA |  |
| LJ124-02 | Sweet orange | Comoros | Mayotte | 2012 | 2518 | 1 | NA |  |
| LJ124-03 | Sweet orange | Comoros | Mayotte | 2012 | 2518 | 1 | NA |  |
| LJ124-04 | Sweet orange | Comoros | Mayotte | 2012 | 2515 | 1 | NA |  |
| LJ125-01 ^c^ | Sweet orange | Comoros | Mayotte | 2012 | 2519 | 1 | NA |  |
| LJ125-02 | Sweet orange | Comoros | Mayotte | 2012 | 2519 | 1 | NA |  |
| LJ125-03 | Sweet orange | Comoros | Mayotte | 2012 | 2520 | 1 | NA |  |
| LJ125-04 | Sweet orange | Comoros | Mayotte | 2012 | 2520 | 1 | NA |  |
| LJ125-05 | Sweet orange | Comoros | Mayotte | 2012 | 2519 | 1 | NA |  |
| LJ125-06 | Sweet orange | Comoros | Mayotte | 2012 | 2519 | 1 | NA |  |
| LJ125-07 | Sweet orange | Comoros | Mayotte | 2012 | 2519 | 1 | NA |  |
| LJ125-08 | Sweet orange | Comoros | Mayotte | 2012 | 2519 | 1 | NA |  |
| LJ125-09 | Sweet orange | Comoros | Mayotte | 2012 | 2520 | 1 | NA |  |
| LJ125-10 | Sweet orange | Comoros | Mayotte | 2012 | 2520 | 1 | NA |  |
| LJ125-11 | Sweet orange | Comoros | Mayotte | 2012 | 2521 | 1 | NA |  |
| LJ125-12 | Sweet orange | Comoros | Mayotte | 2012 | 2521 | 1 | NA |  |
| LJ125-13 | Sweet orange | Comoros | Mayotte | 2012 | 2519 | 1 | NA |  |
| LJ125-14 | Sweet orange | Comoros | Mayotte | 2012 | 2520 | 1 | NA |  |
| LJ125-15 | Sweet orange | Comoros | Mayotte | 2012 | 2519 | 1 | NA |  |
| LJ125-16 | Sweet orange | Comoros | Mayotte | 2012 | 2522 | 1 | NA |  |
| LJ225-01 | Sweet orange | Comoros | Mayotte | 2012 | 2523 | 1 | 2 | 1 |
| LJ225-02 | Sweet orange | Comoros | Mayotte | 2012 | 2524 | 1 | 2 | 1 |
| LJ225-03 | Mexican lime | Comoros | Mayotte | 2012 | 2523 | 1 | 2 | 1 |
| LJ225-04 | Mexican lime | Comoros | Mayotte | 2012 | 2525 | 1 | 2 | 1 |
| LJ226-01 | Sweet orange | Comoros | Mayotte | 2012 | 2526 | 1 | 184 | 1 |
| LJ226-02 ^c^ | Sweet orange | Comoros | Mayotte | 2012 | 2527 | 1 | 2 | 1 |
| LJ226-03 | Sweet orange | Comoros | Mayotte | 2012 | 2528 | 1 | 184 | 1 |
| LJ226-04 | Sweet orange | Comoros | Mayotte | 2012 | 2529 | 1 | 2 | 1 |
| LJ226-05 | Sweet orange | Comoros | Mayotte | 2012 | 2526 | 1 | 184 | 1 |
| LJ227-01 | Sweet orange | Comoros | Mayotte | 2012 | 2530 | 1 | NA |  |
| LJ227-02 | Sweet orange | Comoros | Mayotte | 2012 | 2531 | 1 | 2 | 1 |
| LJ227-03 | Clementine mandarin | Comoros | Mayotte | 2012 | 2532 | 1 | 2 | 1 |
| LJ227-04 | Sweet orange | Comoros | Mayotte | 2012 | 2533 | 1 | 2 | 1 |
| LJ227-05 | Clementine mandarin | Comoros | Mayotte | 2012 | 2534 | 1 | 2 | 1 |
| LJ227-06 | Sweet orange | Comoros | Mayotte | 2012 | 2535 | 1 | 2 | 1 |
| LJ228-01 | Makrut lime | Comoros | Mayotte | 2012 | 2536 | 1 | 2 | 1 |
| LJ229-01 | Tangelo | Comoros | Mayotte | 2012 | 2537 | 1 | 2 | 1 |
| LJ229-02 | Tangelo | Comoros | Mayotte | 2012 | 2537 | 1 | 2 | 1 |
| LJ229-03 | Mandarin | Comoros | Mayotte | 2012 | 2538 | 1 | 2 | 1 |
| LJ229-04 | Citrange | Comoros | Mayotte | 2012 | 2537 | 1 | 2 | 1 |
| LJ229-05 | Mandarin | Comoros | Mayotte | 2012 | 2539 | 1 | 2 | 1 |
| LJ229-06 | Mandarin | Comoros | Mayotte | 2012 | 2537 | 1 | 2 | 1 |
| LJ229-07 | Tangelo | Comoros | Mayotte | 2012 | 2537 | 1 | 2 | 1 |
| LJ229-08 | Grapefruit | Comoros | Mayotte | 2012 | 2540 | 1 | 2 | 1 |
| LJ229-09 | Mandarin | Comoros | Mayotte | 2012 | 2537 | 1 | 2 | 1 |
| LJ229-10 | Citrange | Comoros | Mayotte | 2012 | 2541 | 1 | 2 | 1 |
| LJ229-11 | Tahiti lime | Comoros | Mayotte | 2012 | 2537 | 1 | 2 | 1 |
| LJ229-12 | Grapefruit | Comoros | Mayotte | 2012 | 2542 | 1 | 2 | 1 |
| LJ229-13 | Grapefruit | Comoros | Mayotte | 2012 | 2537 | 1 | 2 | 1 |
| LJ229-14 | Tangelo | Comoros | Mayotte | 2012 | 2537 | 1 | 2 | 1 |
| LJ229-15 | Tangelo | Comoros | Mayotte | 2012 | 2543 | 1 | 2 | 1 |
| LJ229-16 | Tahiti lime | Comoros | Mayotte | 2012 | 2537 | 1 | 2 | 1 |
| LJ230-01 | Sweet orange | Comoros | Mayotte | 2012 | 2544 | 1 | 2 | 1 |
| LJ230-02 | Sweet orange | Comoros | Mayotte | 2012 | 2545 | 1 | 2 | 1 |
| LJ230-03 | Sweet orange | Comoros | Mayotte | 2012 | 2546 | 1 | 2 | 1 |
| LJ230-04 | Sweet orange | Comoros | Mayotte | 2012 | 2547 | 1 | 2 | 1 |
| LJ230-05 | Sweet orange | Comoros | Mayotte | 2012 | 2547 | 1 | 2 | 1 |
| LJ230-06 | Sweet orange | Comoros | Mayotte | 2012 | 2547 | 1 | 2 | 1 |
| LJ230-07 | Sweet orange | Comoros | Mayotte | 2012 | 2548 | 1 | 2 | 1 |
| LJ230-08 | Sweet orange | Comoros | Mayotte | 2012 | 2549 | 1 | 2 | 1 |
| LJ230-09 | Sweet orange | Comoros | Mayotte | 2012 | 2547 | 1 | 2 | 1 |
| LJ231-01 | Sweet orange | Comoros | Mayotte | 2012 | 2550 | 1 | 2 | 1 |
| LJ231-02 | Sweet orange | Comoros | Mayotte | 2012 | 2551 | 1 | 2 | 1 |
| LJ231-03 | Tangelo | Comoros | Mayotte | 2012 | 2533 | 1 | 2 | 1 |
| LJ231-04 | Tahiti lime | Comoros | Mayotte | 2012 | 2531 | 1 | 2 | 1 |
| LJ231-05 | Makrut lime | Comoros | Mayotte | 2012 | 2552 | 1 | 2 | 1 |
| LJ231-06 | Sweet orange | Comoros | Mayotte | 2012 | 2553 | 1 | 2 | 1 |
| LJ231-07 | Tangelo | Comoros | Mayotte | 2012 | 2554 | 1 | 2 | 1 |
| LJ231-08 | Citrange | Comoros | Mayotte | 2012 | 2533 | 1 | 2 | 1 |
| LJ231-09 | *Citrus* sp. | Comoros | Mayotte | 2012 | 2555 | 1 | 2 | 1 |
| LJ231-10 | Sweet orange | Comoros | Mayotte | 2012 | 2533 | 1 | 2 | 1 |
| LJ232-01 | Sweet orange | Comoros | Mayotte | 2012 | 2556 | 1 | 2 | 1 |
| LJ232-02 | Sweet orange | Comoros | Mayotte | 2012 | 2557 | 1 | 2 | 1 |
| LJ232-03 | Sweet orange | Comoros | Mayotte | 2012 | 2558 | 1 | 2 | 1 |
| LJ232-04 | Sweet orange | Comoros | Mayotte | 2012 | 2559 | 1 | 2 | 1 |
| LJ232-05 | Sweet orange | Comoros | Mayotte | 2012 | 2560 | 1 | 2 | 1 |
| LJ232-06 | Sweet orange | Comoros | Mayotte | 2012 | 2561 | 1 | 2 | 1 |
| LJ232-07 | Sweet orange | Comoros | Mayotte | 2012 | 2562 | 1 | 2 | 1 |
| LJ319 | *Citrus* sp. | Comoros | Mayotte | 2012 | 2563 | 1 | NA |  |
| LJ320-1 | *Citrus* sp. | Comoros | Mayotte | 2012 | 2564 | 1 | NA |  |
| LJ320-2 | *Citrus* sp. | Comoros | Mayotte | 2012 | 2565 | 1 | NA |  |
| LJ321-1 | Makrut lime | Comoros | Mayotte | 2012 | 2566 | 1 | NA |  |
| LJ321-2 | Makrut lime | Comoros | Mayotte | 2012 | 2567 | 1 | NA |  |
| LJ321-3 | Makrut lime | Comoros | Mayotte | 2012 | 2568 | 1 | NA |  |
| LJ321-4 | Makrut lime | Comoros | Mayotte | 2012 | 2569 | 1 | NA |  |
| LJ321-5 | Makrut lime | Comoros | Mayotte | 2012 | 2566 | 1 | NA |  |
| LJ321-6 | Makrut lime | Comoros | Mayotte | 2012 | 2566 | 1 | NA |  |
| LJ321-7 | Makrut lime | Comoros | Mayotte | 2012 | 2566 | 1 | NA |  |
| LJ321-8 | Makrut lime | Comoros | Mayotte | 2012 | 2570 | 1 | NA |  |
| LJ321-9 | Makrut lime | Comoros | Mayotte | 2012 | 2566 | 1 | NA |  |
| JJ009-01 | Citrange | Mascarene | Mauritius | 1984 | 2810 | 1 | 35 | 1 |
| JJ009-02 | Mandarin | Mascarene | Mauritius | 1984 | 2811 | 1 | 35 | 1 |
| JJ009-03 | *Citrus* sp. | Mascarene | Mauritius | 1984 | 2812 | m19 | 169 | 1 |
| JJ009-04 | Bergamot orange | Mascarene | Mauritius | 1985 | 2813 | 1 | 4 | 1 |
| JJ009-05 | Lemon | Mascarene | Mauritius | 1985 | 1441 | 1 | NA |  |
| JJ009-08 | Sweet orange | Mascarene | Mauritius | 1987 | 2815 | 1 | 35 | 1 |
| **JJ238-41 ^c^** | ***Citrus* sp.** | **Mascarene** | **Mauritius** | **< 1989** | **AS0606** | **sing.** | **163** | **4** |
| JJ238-42 | *Citrus* sp. | Mascarene | Mauritius | < 1989 | 2821 | 1 | NA |  |
| JK161 ^c^ | Sweet orange | Mascarene | Mauritius | 1990 | 1628 | 1 | 35 | 1 |
| JK163-01 | Tahiti lime | Mascarene | Mauritius | 1990 | 2822 | 1 | 35 | 1 |
| JK163-02 | Tahiti lime | Mascarene | Mauritius | 1990 | 1628 | 1 | 35 | 1 |
| JK164 | Mexican lime | Mascarene | Mauritius | 1990 | 2823 | 1 | 35 | 1 |
| JK165 | Sour orange | Mascarene | Mauritius | 1990 | 2824 | 1 | 51 | 1 |
| JK166 | *Citrus* sp. | Mascarene | Mauritius | 1990 | 2825 | 1 | NA |  |
| JK167-01 | Mexican lime | Mascarene | Mauritius | 1990 | 2815 | 1 | 35 | 1 |
| JK167-02 | Mexican lime | Mascarene | Mauritius | 1990 | 2810 | 1 | NA |  |
| JK168 | *Citrus* sp. | Mascarene | Mauritius | 1990 | 2826 | 1 | 35 | 1 |
| JK169 | Mexican lime | Mascarene | Mauritius | 1990 | 2827 | 1 | 2 | 1 |
| JK170 | Mexican lime | Mascarene | Mauritius | 1990 | 2828 | 1 | 161 | 1 |
| JK172 | Volkamer lemon | Mascarene | Mauritius | 1990 | 2822 | 1 | NA |  |
| JK173 | Mandarin | Mascarene | Mauritius | 1990 | 2829 | 1 | 35 | 1 |
| JK174 | Mandarin | Mascarene | Mauritius | 1990 | 2830 | 1 | 35 | 1 |
| JK175 | Mandarin | Mascarene | Mauritius | 1990 | 2831 | 1 | NA |  |
| JK176-01 | Grapefruit | Mascarene | Mauritius | 1990 | 2832 | m12 | NA |  |
| JK176-02 | Grapefruit | Mascarene | Mauritius | 1990 | 2833 | 1 | NA |  |
| JK178 | Mexican lime | Mascarene | Mauritius | 1990 | 2834 | 1 | NA |  |
| JK179 | *Citrus* sp. | Mascarene | Mauritius | 1990 | 2834 | 1 | NA |  |
| LM053-01 ^c^ | Clementine mandarin | Mascarene | Mauritius | 2015 | 2717 | 1 | 100 | 1 |
| LM053-02 | Clementine mandarin | Mascarene | Mauritius | 2015 | 2718 | 1 | NA |  |
| LM053-03 | Clementine mandarin | Mascarene | Mauritius | 2015 | 2719 | 1 | NA |  |
| LM053-04 | Clementine mandarin | Mascarene | Mauritius | 2015 | 2720 | 1 | NA |  |
| LM053-05 | Mexican lime | Mascarene | Mauritius | 2015 | 2721 | 1 | NA |  |
| LM053-06 | Mexican lime | Mascarene | Mauritius | 2015 | 2722 | 1 | NA |  |
| LM053-07 | Mexican lime | Mascarene | Mauritius | 2015 | 2723 | 1 | NA |  |
| LM053-08 | Mexican lime | Mascarene | Mauritius | 2015 | 2724 | 1 | NA |  |
| LM053-09 | Mexican lime | Mascarene | Mauritius | 2015 | 2725 | 1 | 11 | 1 |
| LM053-10 | Mexican lime | Mascarene | Mauritius | 2015 | 2726 | 1 | NA |  |
| LM053-11 | Mexican lime | Mascarene | Mauritius | 2015 | 2720 | 1 | NA |  |
| LM053-12 | Mexican lime | Mascarene | Mauritius | 2015 | 2717 | 1 | NA |  |
| LM053-13 | Mexican lime | Mascarene | Mauritius | 2015 | 2721 | 1 | NA |  |
| LM053-14 | Mexican lime | Mascarene | Mauritius | 2015 | 2727 | 1 | NA |  |
| **LM053-15 ^c^** | **Mexican lime** | **Mascarene** | **Mauritius** | **2015** | **AS0225** | **sing.** | **220** | **4** |
| LM053-16 | Mexican lime | Mascarene | Mauritius | 2015 | 2720 | 1 | NA |  |
| LM053-17 | Mexican lime | Mascarene | Mauritius | 2015 | 2727 | 1 | NA |  |
| LM053-18 | Mexican lime | Mascarene | Mauritius | 2015 | 2717 | 1 | NA |  |
| LM053-19 | Mexican lime | Mascarene | Mauritius | 2015 | 2721 | 1 | NA |  |
| LM053-20 | Mexican lime | Mascarene | Mauritius | 2015 | 2727 | 1 | NA |  |
| LM054-01 | Sweet orange | Mascarene | Mauritius | 2015 | 2729 | 1 | 12 | 1 |
| LM054-02 | Sweet orange | Mascarene | Mauritius | 2015 | 2730 | 1 | NA |  |
| LM054-03 | Sweet orange | Mascarene | Mauritius | 2015 | 2731 | 1 | NA |  |
| LM054-04 | Sweet orange | Mascarene | Mauritius | 2015 | 2732 | 1 | NA |  |
| LM054-05 | Sweet orange | Mascarene | Mauritius | 2015 | 2733 | 1 | 125 | 1 |
| LM054-06 | Sweet orange | Mascarene | Mauritius | 2015 | 2734 | 1 | NA |  |
| LM054-07 | Sweet orange | Mascarene | Mauritius | 2015 | 2735 | 1 | NA |  |
| LM054-08 | Sweet orange | Mascarene | Mauritius | 2015 | 2734 | 1 | NA |  |
| LM054-09 | Sweet orange | Mascarene | Mauritius | 2015 | 2729 | 1 | NA |  |
| LM054-10 | Sweet orange | Mascarene | Mauritius | 2015 | 2736 | 1 | NA |  |
| LM054-11 | Sweet orange | Mascarene | Mauritius | 2015 | 2729 | 1 | NA |  |
| LM054-12 | Sweet orange | Mascarene | Mauritius | 2015 | 2734 | 1 | NA |  |
| LM054-13 | Sweet orange | Mascarene | Mauritius | 2015 | 2732 | 1 | 35 | 1 |
| LM054-14 | Sweet orange | Mascarene | Mauritius | 2015 | 2737 | 1 | NA |  |
| LM054-15 | Sweet orange | Mascarene | Mauritius | 2015 | 2729 | 1 | NA |  |
| LM054-16 | Sweet orange | Mascarene | Mauritius | 2015 | 2735 | 1 | NA |  |
| LM054-17 | Sweet orange | Mascarene | Mauritius | 2015 | 2738 | 1 | NA |  |
| LM054-18 | Sweet orange | Mascarene | Mauritius | 2015 | 2729 | 1 | NA |  |
| LM054-20 | Sweet orange | Mascarene | Mauritius | 2015 | 2729 | 1 | NA |  |
| LM054-21 | Sweet orange | Mascarene | Mauritius | 2015 | 2737 | 1 | NA |  |
| LM055-01 | Tangelo | Mascarene | Mauritius | 2015 | 2739 | 1 | 11 | 1 |
| LM055-02 | Tangelo | Mascarene | Mauritius | 2015 | 2739 | 1 | NA |  |
| LM055-03 | Tangelo | Mascarene | Mauritius | 2015 | 2740 | 1 | NA |  |
| LM055-04 | Tangelo | Mascarene | Mauritius | 2015 | 2740 | 1 | NA |  |
| LM055-05 | Tangelo | Mascarene | Mauritius | 2015 | 2740 | 1 | NA |  |
| LM055-06 | Tangelo | Mascarene | Mauritius | 2015 | 2740 | 1 | NA |  |
| LM055-07 | Tangelo | Mascarene | Mauritius | 2015 | 2741 | 1 | NA |  |
| LM055-08 | Tangelo | Mascarene | Mauritius | 2015 | 2741 | 1 | 35 | 1 |
| LM055-09 | Tangelo | Mascarene | Mauritius | 2015 | 2741 | 1 | NA |  |
| LM055-10 | Tangelo | Mascarene | Mauritius | 2015 | 2741 | 1 | NA |  |
| LM055-11 | Tangelo | Mascarene | Mauritius | 2015 | 2741 | 1 | NA |  |
| LM055-12 | Tangelo | Mascarene | Mauritius | 2015 | 2741 | 1 | NA |  |
| LM055-13 | Tangelo | Mascarene | Mauritius | 2015 | 2741 | 1 | NA |  |
| LM055-14 | Tangelo | Mascarene | Mauritius | 2015 | 2741 | 1 | NA |  |
| LM055-15 | Tangelo | Mascarene | Mauritius | 2015 | 2741 | 1 | NA |  |
| LM055-16 | Tangelo | Mascarene | Mauritius | 2015 | 2741 | 1 | NA |  |
| LM055-17 | Tangelo | Mascarene | Mauritius | 2015 | 2742 | 1 | NA |  |
| LM055-18 | Tangelo | Mascarene | Mauritius | 2015 | 2741 | 1 | NA |  |
| LM055-19 | Tangelo | Mascarene | Mauritius | 2015 | 2741 | 1 | NA |  |
| LM057-01 | Meyer lemon | Mascarene | Mauritius | 2015 | 2721 | 1 | NA |  |
| LM057-02 | Meyer lemon | Mascarene | Mauritius | 2015 | 2743 | 1 | NA |  |
| LM057-03 | Meyer lemon | Mascarene | Mauritius | 2015 | 2721 | 1 | NA |  |
| LM057-04 | Meyer lemon | Mascarene | Mauritius | 2015 | 2744 | 1 | NA |  |
| LM057-05 | Meyer lemon | Mascarene | Mauritius | 2015 | 2745 | 1 | 35 | 1 |
| LM057-06 | Meyer lemon | Mascarene | Mauritius | 2015 | 2745 | 1 | NA |  |
| LM057-07 | Meyer lemon | Mascarene | Mauritius | 2015 | 2745 | 1 | NA |  |
| LM057-08 | Meyer lemon | Mascarene | Mauritius | 2015 | 2746 | 1 | NA |  |
| LM057-09 | Meyer lemon | Mascarene | Mauritius | 2015 | 2745 | 1 | NA |  |
| LM057-10 | Meyer lemon | Mascarene | Mauritius | 2015 | 2742 | 1 | NA |  |
| LM057-11 | Meyer lemon | Mascarene | Mauritius | 2015 | 2742 | 1 | NA |  |
| LM057-12 | Meyer lemon | Mascarene | Mauritius | 2015 | 2721 | 1 | NA |  |
| LM057-13 | Meyer lemon | Mascarene | Mauritius | 2015 | 2747 | 1 | NA |  |
| LM057-14 | Meyer lemon | Mascarene | Mauritius | 2015 | 2748 | 1 | NA |  |
| LM057-15 | Meyer lemon | Mascarene | Mauritius | 2015 | 2749 | 1 | NA |  |
| LM057-16 | Meyer lemon | Mascarene | Mauritius | 2015 | 2750 | 1 | NA |  |
| LM058-01 | Bergamot orange | Mascarene | Mauritius | 2015 | 2751 | 1 | NA |  |
| LM058-02 | Bergamot orange | Mascarene | Mauritius | 2015 | 2752 | 1 | NA |  |
| LM069-01 | Mexican lime | Mascarene | Mauritius | 2015 | 2753 | sing. | 124 | 1 |
| LM069-02 | Mexican lime | Mascarene | Mauritius | 2015 | 2753 | sing. | NA |  |
| LM070 | Mexican lime | Mascarene | Mauritius | 2015 | 2754 | m10 | NA |  |
| LM071 | Mexican lime | Mascarene | Mauritius | 2015 | 2755 | 1 | NA |  |
| LM072 | Mandarin | Mascarene | Mauritius | 2015 | 2756 | 1 | 4 | 1 |
| LM073-01 | *Citru*s sp. | Mascarene | Mauritius | 2015 | 2757 | 1 | NA |  |
| LM073-02 | *Citru*s sp. | Mascarene | Mauritius | 2015 | 2758 | 1 | 4 | 1 |
| LM074 | Lemon | Mascarene | Mauritius | 2015 | 2759 | 1 | 12 | 1 |
| LM075 | Lemon | Mascarene | Mauritius | 2015 | 2760 | 1 | NA |  |
| LM076-01 | Lemon | Mascarene | Mauritius | 2015 | 2761 | 1 | NA |  |
| LM077-01 | Lemon | Mascarene | Mauritius | 2015 | 2762 | 1 | 4 | 1 |
| LM077-02 | Lemon | Mascarene | Mauritius | 2015 | 2762 | 1 | NA |  |
| LM077-03 | Lemon | Mascarene | Mauritius | 2015 | 2762 | 1 | NA |  |
| LM078-01 | Lemon | Mascarene | Mauritius | 2015 | 2763 | 1 | NA |  |
| LM078-02 | Lemon | Mascarene | Mauritius | 2015 | 2763 | 1 | NA |  |
| LM079 | Lemon | Mascarene | Mauritius | 2015 | 2764 | 1 | NA |  |
| LM080 | Lemon | Mascarene | Mauritius | 2015 | 2765 | 1 | NA |  |
| LM081 | Lemon | Mascarene | Mauritius | 2015 | 2766 | 1 | NA |  |
| LM082-01 | *Citru*s sp. | Mascarene | Mauritius | 2015 | 2767 | 1 | NA |  |
| LM082-02 | *Citru*s sp. | Mascarene | Mauritius | 2015 | 2767 | 1 | NA |  |
| LM083-01 | *Citru*s sp. | Mascarene | Mauritius | 2015 | 2760 | 1 | NA |  |
| LM083-02 | *Citru*s sp. | Mascarene | Mauritius | 2015 | 2760 | 1 | NA |  |
| LM084-01 | *Citru*s sp. | Mascarene | Mauritius | 2015 | 2763 | 1 | NA |  |
| LM084-02 | *Citru*s sp. | Mascarene | Mauritius | 2015 | 2768 | 1 | NA |  |
| LM085 | *Citru*s sp. | Mascarene | Mauritius | 2015 | 2756 | 1 | 161 | 1 |
| LM086-01 | *Citru*s sp. | Mascarene | Mauritius | 2015 | 2769 | 1 | NA |  |
| LM086-02 | *Citru*s sp. | Mascarene | Mauritius | 2015 | 2765 | 1 | NA |  |
| LM086-03 | *Citru*s sp. | Mascarene | Mauritius | 2015 | 2765 | 1 | NA |  |
| LM086-04 | *Citru*s sp. | Mascarene | Mauritius | 2015 | 2765 | 1 | NA |  |
| LM087-01 | *Citru*s sp. | Mascarene | Mauritius | 2015 | 2761 | 1 | NA |  |
| LM087-02 | *Citru*s sp. | Mascarene | Mauritius | 2015 | 2761 | 1 | NA |  |
| JJ010-01 | Mexican lime | Mascarene | Rodrigues | 1985 | 2816 | 1 | 35 | 1 |
| JJ010-02 | Mexican lime | Mascarene | Rodrigues | 1985 | 2817 | sing. | 161 | 1 |
| JJ010-03 | Mexican lime | Mascarene | Rodrigues | 1985 | 2812 | m19 | 169 | 1 |
| JJ010-04 | Mexican lime | Mascarene | Rodrigues | 1985 | 2818 | 1 | 198 | 1 |
| JJ010-05 | Mexican lime | Mascarene | Rodrigues | 1985 | 2819 | 1 | 169 | 1 |
| JJ010-06 | Mexican lime | Mascarene | Rodrigues | 1985 | 2814 | m13 | 161 | 1 |
| JJ010-07 | Mexican lime | Mascarene | Rodrigues | 1985 | 2820 | 1 | 141 | 1 |
| LC043 | Mexican lime | Mascarene | Rodrigues | 2006 | 2497 | 1 | 161 | 1 |
| LC044 | Mexican lime | Mascarene | Rodrigues | 2006 | 2498 | 1 | 169 | 1 |
| LC045-01 | Mexican lime | Mascarene | Rodrigues | 2006 | 2496 | sing. | 170 | 1 |
| LC045-02 | Mexican lime | Mascarene | Rodrigues | 2006 | 2499 | sing. | 171 | 1 |
| LC046 | Mexican lime | Mascarene | Rodrigues | 2006 | 2500 | sing. | 141 | 1 |
| LC048 | Mexican lime | Mascarene | Rodrigues | 2006 | 2501 | sing. | 161 | 1 |
| LC049-01 | Mexican lime | Mascarene | Rodrigues | 2006 | 2502 | m8 | 141 | 1 |
| LC049-02 | Mexican lime | Mascarene | Rodrigues | 2006 | 2503 | m8 | 141 | 1 |
| LC049-03 | Mandarin | Mascarene | Rodrigues | 2006 | 2504 | m8 | 141 | 1 |
| LI139-01 | Mandarin | Mascarene | Rodrigues | 2011 | 2506 | sing. | 161 | 1 |
| LI139-03 | Mandarin | Mascarene | Rodrigues | 2011 | 2507 | sing. | 161 | 1 |
| LI139-04 | Mandarin | Mascarene | Rodrigues | 2011 | 2508 | sing. | 141 | 1 |
| LI139-05 | Mandarin | Mascarene | Rodrigues | 2011 | 2509 | sing. | 199 | 1 |
| LI139-06 | Mandarin | Mascarene | Rodrigues | 2011 | 2510 | 1 | 141 | 1 |
| LI139-07 | Mandarin | Mascarene | Rodrigues | 2011 | 2511 | sing. | 169 | 1 |
| LI139-08 | Mandarin | Mascarene | Rodrigues | 2011 | 2512 | 1 | NA |  |
| LM092 | *Citru*s sp. | Mascarene | Rodrigues | 2015 | 2770 | 1 | NA |  |
| LM093 | Mexican lime | Mascarene | Rodrigues | 2015 | 2771 | 1 | NA |  |
| LM094 | Mandarin | Mascarene | Rodrigues | 2015 | 2772 | 1 | NA |  |
| LM095-01 | Mexican lime | Mascarene | Rodrigues | 2015 | 2773 | 1 | NA |  |
| LM095-02 | Mexican lime | Mascarene | Rodrigues | 2015 | 2774 | m16 | NA |  |
| LM095-03 | Mexican lime | Mascarene | Rodrigues | 2015 | 2773 | 1 | NA |  |
| LM095-04 | Mexican lime | Mascarene | Rodrigues | 2015 | 2775 | m16 | 207 | 1 |
| LM095-05 ^c^ | Mexican lime | Mascarene | Rodrigues | 2015 | 2776 | 1 | NA |  |
| LM095-06 | Mexican lime | Mascarene | Rodrigues | 2015 | 2773 | 1 | NA |  |
| LM095-07 | Mexican lime | Mascarene | Rodrigues | 2015 | 2777 | sing. | 211 | 1 |
| LM095-08 | Mexican lime | Mascarene | Rodrigues | 2015 | 2777 | sing. | NA |  |
| LM095-09 | Mexican lime | Mascarene | Rodrigues | 2015 | 2778 | 1 | NA |  |
| LM095-10 | Mexican lime | Mascarene | Rodrigues | 2015 | 2779 | 1 | NA |  |
| LM095-11 | Mexican lime | Mascarene | Rodrigues | 2015 | 2780 | sing. | 212 | 1 |
| LM095-12 | Mexican lime | Mascarene | Rodrigues | 2015 | 2781 | 1 | NA |  |
| LM095-13 | Mexican lime | Mascarene | Rodrigues | 2015 | 2773 | 1 | NA |  |
| LM095-14 | Mexican lime | Mascarene | Rodrigues | 2015 | 2782 | sing. | 200 | 1 |
| LM095-15 | Mexican lime | Mascarene | Rodrigues | 2015 | 2783 | 1 | 141 | 1 |
| LM095-16 | Mexican lime | Mascarene | Rodrigues | 2015 | 2784 | 1 | NA |  |
| LM095-17 | Mexican lime | Mascarene | Rodrigues | 2015 | 2785 | 1 | NA |  |
| LM095-18 | Mexican lime | Mascarene | Rodrigues | 2015 | 2786 | 1 | NA |  |
| LM095-19 | Mexican lime | Mascarene | Rodrigues | 2015 | 2787 | 1 | NA |  |
| LM095-20 | Mexican lime | Mascarene | Rodrigues | 2015 | 2787 | 1 | NA |  |
| LM096-01 | Mexican lime | Mascarene | Rodrigues | 2015 | 2788 | 1 | NA |  |
| LM096-02 | Mexican lime | Mascarene | Rodrigues | 2015 | 2789 | 1 | NA |  |
| LM096-03 | Mexican lime | Mascarene | Rodrigues | 2015 | 2790 | sing. | 200 | 1 |
| LM096-04 | Mexican lime | Mascarene | Rodrigues | 2015 | 2791 | 1 | NA |  |
| LM096-05 | Mexican lime | Mascarene | Rodrigues | 2015 | 2792 | 1 | 33 | 1 |
| LM096-06 | Mexican lime | Mascarene | Rodrigues | 2015 | 2793 | 1 | NA |  |
| LM096-07 | Mexican lime | Mascarene | Rodrigues | 2015 | 2793 | 1 | NA |  |
| LM096-08 | Mexican lime | Mascarene | Rodrigues | 2015 | 2794 | sing. | 207 | 1 |
| LM096-09 | Mexican lime | Mascarene | Rodrigues | 2015 | 2795 | sing. | 215 | 1 |
| LM096-10 | Mexican lime | Mascarene | Rodrigues | 2015 | 2796 | 1 | NA |  |
| LM097-01 | Mexican lime | Mascarene | Rodrigues | 2015 | 2797 | 1 | NA |  |
| LM097-02 | Mexican lime | Mascarene | Rodrigues | 2015 | 2798 | 1 | NA |  |
| LM097-03 | Mexican lime | Mascarene | Rodrigues | 2015 | 2797 | 1 | NA |  |
| LM097-04 | Mexican lime | Mascarene | Rodrigues | 2015 | 2797 | 1 | NA |  |
| LM097-05 | Mexican lime | Mascarene | Rodrigues | 2015 | 2799 | 1 | NA |  |
| LM097-06 | Mexican lime | Mascarene | Rodrigues | 2015 | 2800 | 1 | NA |  |
| LM097-07 | Mexican lime | Mascarene | Rodrigues | 2015 | 2799 | 1 | NA |  |
| LM097-08 | Mexican lime | Mascarene | Rodrigues | 2015 | 2801 | 1 | NA |  |
| LM098-01 | Mexican lime | Mascarene | Rodrigues | 2015 | 2802 | 1 | NA |  |
| LM098-02 | Mexican lime | Mascarene | Rodrigues | 2015 | 2803 | 1 | NA |  |
| LM098-03 | Mexican lime | Mascarene | Rodrigues | 2015 | 2804 | 1 | NA |  |
| LM098-04 | Mexican lime | Mascarene | Rodrigues | 2015 | 2805 | 1 | 177 | 1 |
| LM099-01 | Mexican lime | Mascarene | Rodrigues | 2015 | 2806 | sing. | 216 | 1 |
| LM099-02 | Mexican lime | Mascarene | Rodrigues | 2015 | 2806 | sing. | NA |  |
| LM099-03 | Mexican lime | Mascarene | Rodrigues | 2015 | 2807 | sing. | 214 | 1 |
| LM099-04 | Mexican lime | Mascarene | Rodrigues | 2015 | 2808 | 1 | NA |  |
| LM099-05 | Mexican lime | Mascarene | Rodrigues | 2015 | 2809 | 1 | 184 | 1 |
| JZ089 ^c^ | *Citrus* sp. | Seychelles | Mahé | 2003 | 2486 | 2 | 168 | 1 |
| JZ090 | Sweet orange | Seychelles | Mahé | 2003 | 2487 | 2 | 168 | 1 |
| JZ091 | Sweet orange | Seychelles | Mahé | 2003 | 2487 | 2 | 168 | 1 |
| JZ092 | Lime | Seychelles | Mahé | 2003 | 2487 | 2 | 168 | 1 |
| JZ093 ^c^ | Lime | Seychelles | Mahé | 2003 | 2488 | 2 | 168 | 1 |
| JZ094 ^c^ | Lime | Seychelles | Mahé | 2003 | 2489 | 3 | 11 | 1 |
| LB100-01 | Citrange | Seychelles | Mahé | 2005 | 2492 | 2 | 168 | 1 |
| LB100-02 | Citrange | Seychelles | Mahé | 2005 | 2493 | 2 | NA |  |
| LB100-03 | Citrange | Seychelles | Mahé | 2005 | 2494 | 2 | 168 | 1 |
| LB100-04 | Citrange | Seychelles | Mahé | 2005 | 2495 | 2 | NA |  |
| LJ001 ^c^ | Sweet orange | Seychelles | Mahé | 2012 | 2513 | 2 | 168 | 1 |
| LJ002-1 | Sweet orange | Seychelles | Mahé | 2012 | 2514 | 2 | NA |  |
| LJ002-2 | Sweet orange | Seychelles | Mahé | 2012 | 2514 | 2 | NA |  |
| LJ003-1 ^c^ | Sweet orange | Seychelles | Mahé | 2012 | 2514 | 2 | 168 | 1 |
| LJ003-2 | Sweet orange | Seychelles | Mahé | 2012 | 2514 | 2 | NA |  |
| LJ004 | Sweet orange | Seychelles | Mahé | 2012 | 2514 | 2 | NA |  |
| LP026-01 ^c^ | Mexican lime | Seychelles | Mahé | 2017 | 2923 | 2 | 217 | 1 |
| LP026-02 | Mexican lime | Seychelles | Mahé | 2017 | 2924 | 2 | NA |  |
| LP026-03 | Mexican lime | Seychelles | Mahé | 2017 | 2923 | 2 | NA |  |
| LP026-04 | Mexican lime | Seychelles | Mahé | 2017 | 2924 | 2 | NA |  |
| LP026-05 ^c^ | Mexican lime | Seychelles | Mahé | 2017 | 2925 | 2 | NA |  |
| LP026-06 | Mexican lime | Seychelles | Mahé | 2017 | 2924 | 2 | NA |  |
| LP026-07 | Mexican lime | Seychelles | Mahé | 2017 | 2926 | 2 | NA |  |
| LP027-01 ^c^ | Mexican lime | Seychelles | Mahé | 2017 | 2838 | 3 | NA |  |
| LP027-02 ^c^ | Mexican lime | Seychelles | Mahé | 2017 | 2927 | 3 | NA |  |
| LP027-03 | Mexican lime | Seychelles | Mahé | 2017 | 2835 | 3 | 218 | 1 |
| LP027-04 | Mexican lime | Seychelles | Mahé | 2017 | 2836 | 3 | NA |  |
| LP027-05 ^c^ | Mexican lime | Seychelles | Mahé | 2017 | 2836 | 3 | 208 | 1 |
| LP027-06 ^c^ | Mexican lime | Seychelles | Mahé | 2017 | 2928 | 3 | NA |  |
| LP027-07 | Mexican lime | Seychelles | Mahé | 2017 | 2837 | 3 | NA |  |
| LP027-08 | Mexican lime | Seychelles | Mahé | 2017 | 2838 | 3 | NA |  |
| LP027-09 ^c^ | Mexican lime | Seychelles | Mahé | 2017 | 2839 | 3 | NA |  |
| LP027-10 | Mexican lime | Seychelles | Mahé | 2017 | 2837 | 3 | NA |  |
| LP027-11 | Mexican lime | Seychelles | Mahé | 2017 | 2835 | 3 | NA |  |
| LP027-12 ^c^ | Mexican lime | Seychelles | Mahé | 2017 | 2840 | 3 | NA |  |
| LP027-13 ^c^ | Mexican lime | Seychelles | Mahé | 2017 | 2841 | 3 | 208 | 1 |
| LP027-14 | Mexican lime | Seychelles | Mahé | 2017 | 2842 | 3 | NA |  |
| LP027-15 | Mexican lime | Seychelles | Mahé | 2017 | 2836 | 3 | 219 | 1 |
| LP027-16 | Mexican lime | Seychelles | Mahé | 2017 | 2836 | 3 | NA |  |
| LP027-17 | Mexican lime | Seychelles | Mahé | 2017 | 2835 | 3 | NA |  |
| LP027-18 | Mexican lime | Seychelles | Mahé | 2017 | 2836 | 3 | NA |  |
| LP027-19 | Mexican lime | Seychelles | Mahé | 2017 | 2836 | 3 | NA |  |
| LP027-20 | Mexican lime | Seychelles | Mahé | 2017 | 2843 | 3 | NA |  |
| LP028-01 ^c^ | Mexican lime | Seychelles | Mahé | 2017 | 2845 | 4 | NA |  |
| LP028-02 ^c^ | Mexican lime | Seychelles | Mahé | 2017 | 2929 | 4 | 209 | 1 |
| LP028-03 ^c^ | Mexican lime | Seychelles | Mahé | 2017 | 2844 | 4 | 209 | 1 |
| LP028-04 | Mexican lime | Seychelles | Mahé | 2017 | 2844 | 4 | NA |  |
| LP028-05 ^c^ | Mexican lime | Seychelles | Mahé | 2017 | 2845 | 4 | 209 | 1 |
| LP028-06 ^c^ | Mexican lime | Seychelles | Mahé | 2017 | 2846 | 4 | 209 | 1 |
| LP028-08 ^c^ | Mexican lime | Seychelles | Mahé | 2017 | 2847 | 4 | NA |  |
| LP028-09 ^c^ | Mexican lime | Seychelles | Mahé | 2017 | 2930 | 4 | NA |  |
| LP028-11 ^c^ | Mexican lime | Seychelles | Mahé | 2017 | 2931 | 4 | NA |  |
| LP028-12 | Mexican lime | Seychelles | Mahé | 2017 | 2844 | 4 | NA |  |
| LP028-13 | Mexican lime | Seychelles | Mahé | 2017 | 2844 | 4 | NA |  |
| LP028-14 ^c^ | Mexican lime | Seychelles | Mahé | 2017 | 2932 | 4 | 209 | 1 |
| LP028-15 | Mexican lime | Seychelles | Mahé | 2017 | 2933 | 4 | NA |  |
| LP028-16 | Mexican lime | Seychelles | Mahé | 2017 | 2835 | 4 | NA |  |
| LP028-17 | Mexican lime | Seychelles | Mahé | 2017 | 2934 | 4 | NA |  |
| LP028-18 ^c^ | Mexican lime | Seychelles | Mahé | 2017 | 2935 | 4 | 208 | 1 |
| LP028-19 | Mexican lime | Seychelles | Mahé | 2017 | 2936 | 4 | NA |  |
| LP029-01 | Sweet orange | Seychelles | Mahé | 2017 | 2851 | 2 | NA |  |
| LP029-02 | Sweet orange | Seychelles | Mahé | 2017 | 2848 | 2 | NA |  |
| LP029-03 | Sweet orange | Seychelles | Mahé | 2017 | 2848 | 2 | NA |  |
| LP029-04 | Sweet orange | Seychelles | Mahé | 2017 | 2937 | 2 | NA |  |
| LP029-05 | Sweet orange | Seychelles | Mahé | 2017 | 2851 | 2 | NA |  |
| LP029-06 | Sweet orange | Seychelles | Mahé | 2017 | 2848 | 2 | NA |  |
| LP029-07 | Sweet orange | Seychelles | Mahé | 2017 | 2848 | 2 | NA |  |
| LP029-08 | Sweet orange | Seychelles | Mahé | 2017 | 2848 | 2 | NA |  |
| LP029-09 | Sweet orange | Seychelles | Mahé | 2017 | 2848 | 2 | NA |  |
| LP029-10 ^c^ | Sweet orange | Seychelles | Mahé | 2017 | 2849 | 2 | NA |  |
| LP029-11 | Sweet orange | Seychelles | Mahé | 2017 | 2938 | 2 | 210 | 1 |
| LP029-12 | Sweet orange | Seychelles | Mahé | 2017 | 2848 | 2 | NA |  |
| LP029-13 | Sweet orange | Seychelles | Mahé | 2017 | 2850 | 2 | 210 | 1 |
| LP029-14 | Sweet orange | Seychelles | Mahé | 2017 | 2939 | 2 | NA |  |
| LP029-15 ^c^ | Sweet orange | Seychelles | Mahé | 2017 | 2851 | 2 | 210 | 1 |
| LP029-16 | Sweet orange | Seychelles | Mahé | 2017 | 2852 | 2 | NA |  |
| LP029-17 | Sweet orange | Seychelles | Mahé | 2017 | 2851 | 2 | NA |  |
| LP029-18 | Sweet orange | Seychelles | Mahé | 2017 | 2851 | 2 | NA |  |
| LP029-19 | Sweet orange | Seychelles | Mahé | 2017 | 2853 | 2 | NA |  |
| LP029-20 | Sweet orange | Seychelles | Mahé | 2017 | 2851 | 2 | NA |  |
| LP029-21 | Sweet orange | Seychelles | Mahé | 2017 | 2851 | 2 | NA |  |

NA: not available.

Bold characters indicate strains that were assigned to lineage 4 by minisatellite genotyping and further identified as pathotype A* by pathogenicity assays.

^a^Numbers preceded by m refer to minor clusters; sing. is for singletons.

^b^According to Pruvost et al. (2014).

^c^Strains assayed with the XAC1051-qPCR.
