# Supplementary figures and images for: Diversity and Geographical Structure of Xanthomonas citri pv. citri on Citrus in the South West Indian Ocean Region
Source: Microorganisms. 2021 Apr 27;9(5):945. doi: 10.3390/microorganisms9050945 (PMC8146439; doi:10.3390/microorganisms9050945)

Strata GC1 GC2 GC3 GC4

Mandarin (*C. reticulata*) cv. Zanzibar

Survival probability

$p = 0.72$

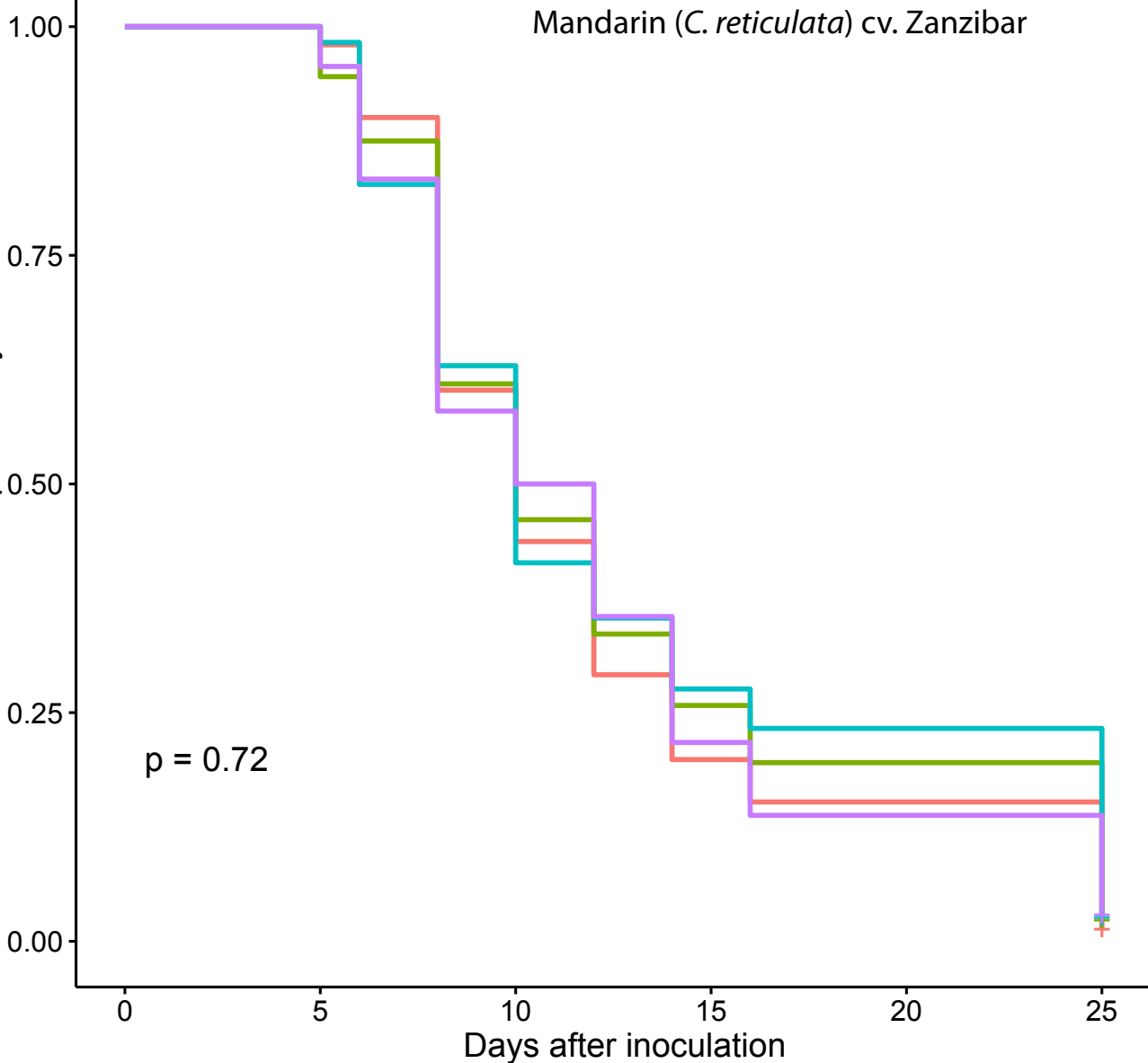

Supplement: Supplementary file 1 [file microorganisms-09-00945-s001.zip › V2/FigS4.pdf]

Strata + GC1 + GC2 + GC3 + GC4

Citron (*C. medica*) cv. Buddha's hand

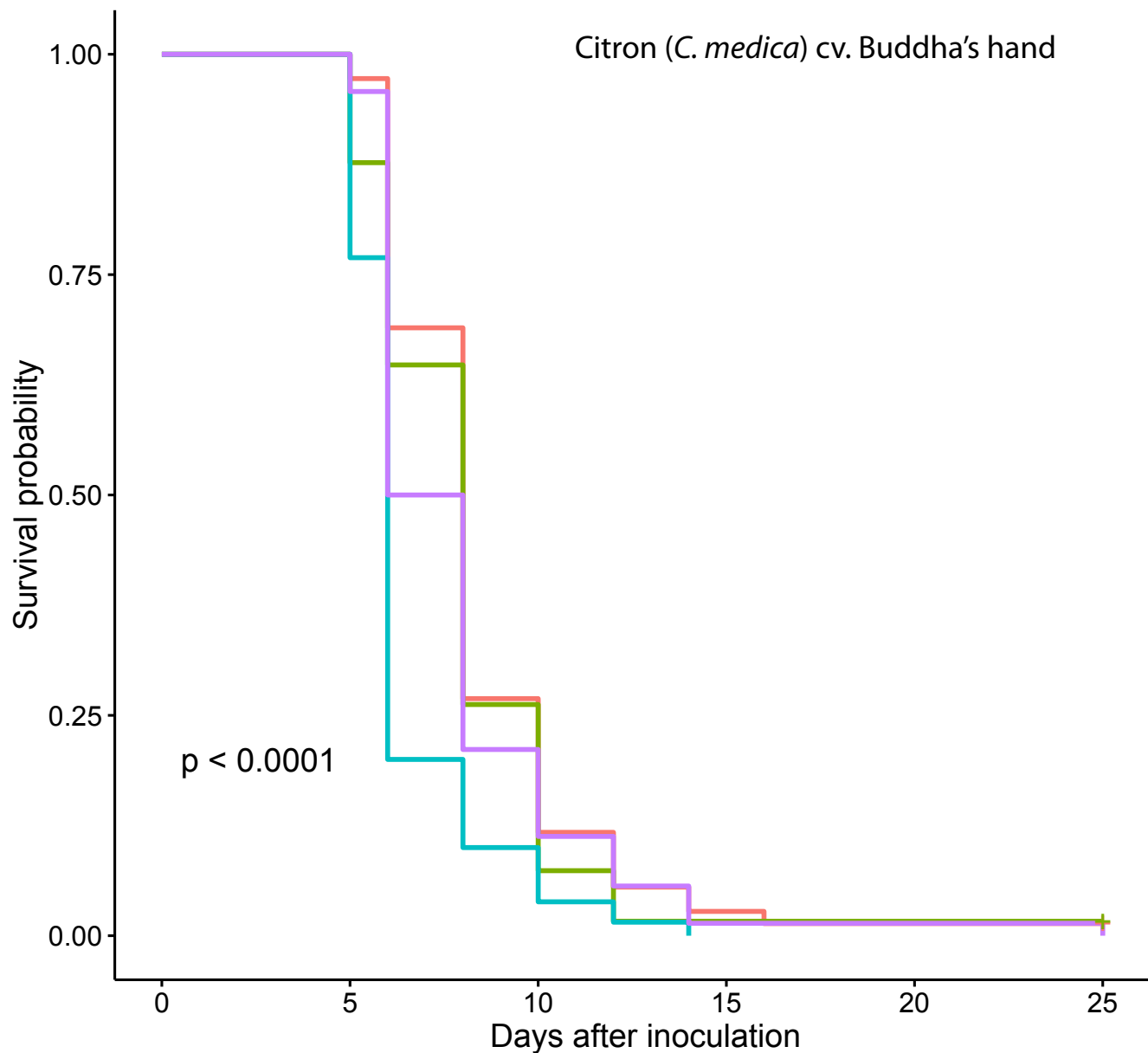

Supplement: Supplementary file 1 [file microorganisms-09-00945-s001.zip › V2/FigS2.pdf]

Strata + GC1 + GC2 + GC3 + GC4

Sweet orange (*C. x sinensis*) cv. New Hall navel

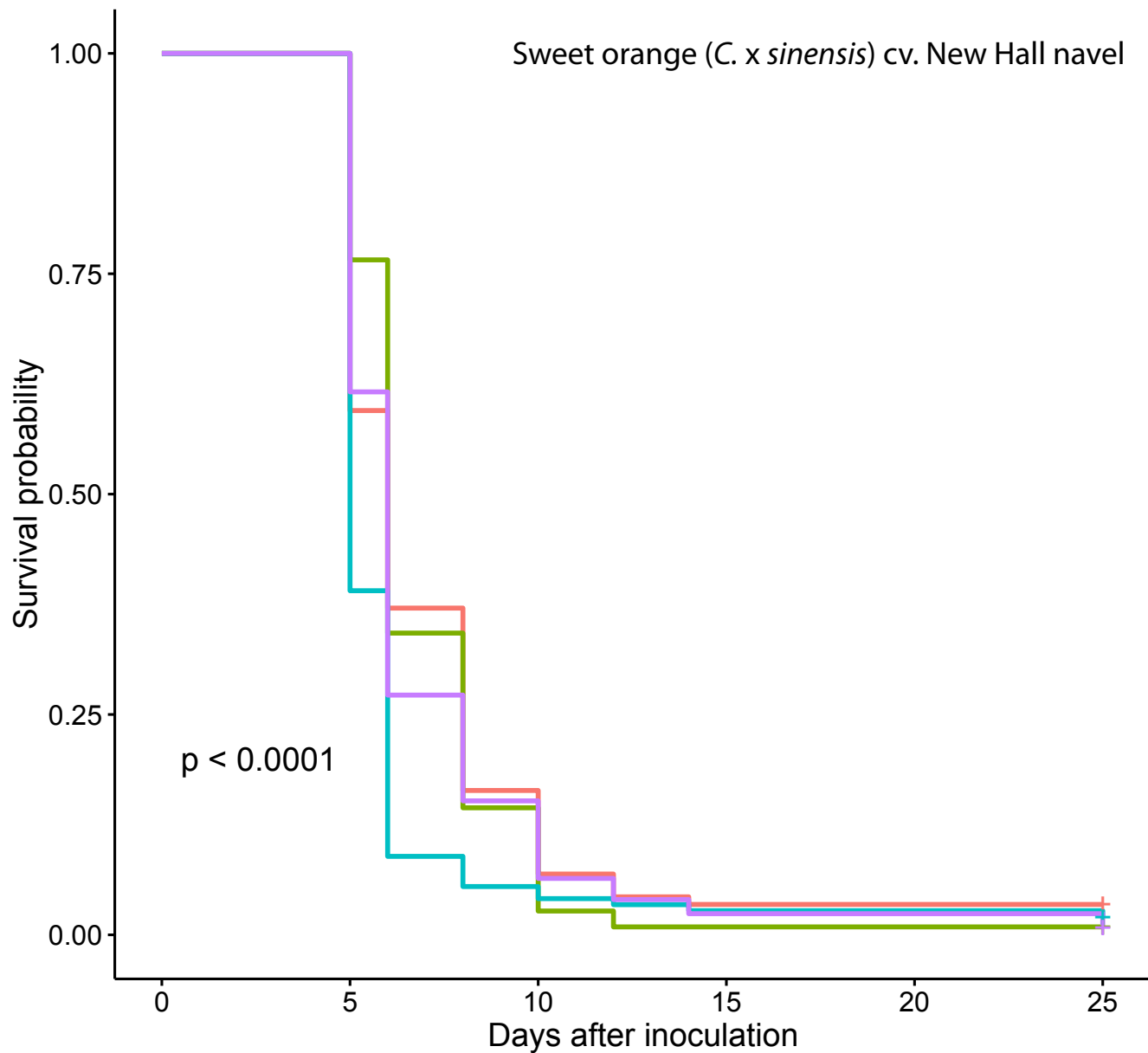

Supplement: Supplementary file 1 [file microorganisms-09-00945-s001.zip › V2/FigS3.pdf]

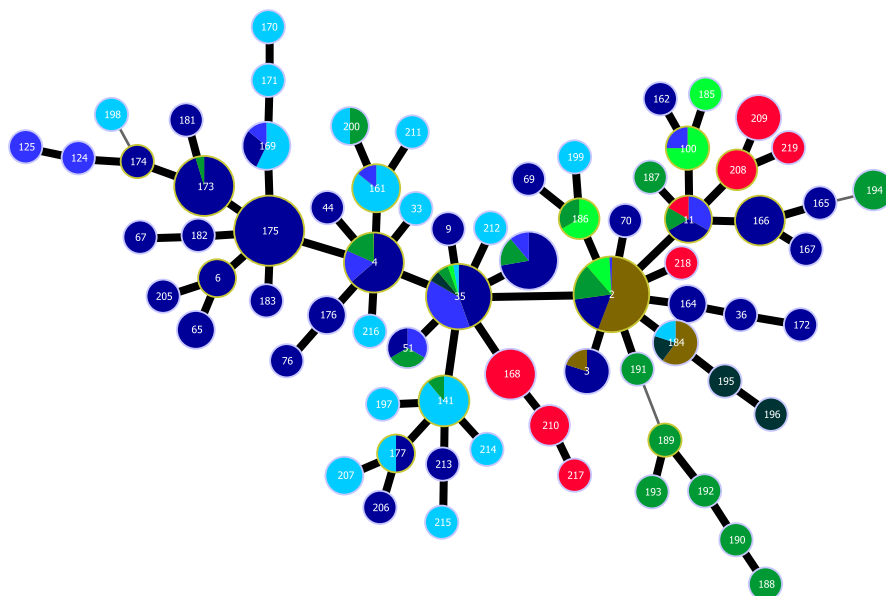

Supplement: Supplementary file 1 [file microorganisms-09-00945-s001.zip › V2/FigS1.pdf]
